# Standoff Time-Resolved SECARS on Ag-Decorated Silicon Nanowires

**DOI:** 10.1021/acsphotonics.6c01317

**Published:** 2026-09-24

**Authors:** Marco Polastri, Antonio Alessio Leonardi, Ileana Ielo, Barbara Fazio, Giulio Cerullo, Alessia Irrera, Nicola Coluccelli

**Affiliations:** † Department of Physics, 18981 Politecnico di Milano, Piazza Leonardo da Vinci 32, Milano 20132, Italy; ‡ Department of Chemical, Biological, Pharmaceutical, and Environmental Sciences (ChiBioFarAm), 18980 University of Messina, Viale F. S. d’Alcontres 31, Messina I-98166, Italy; § 9327 Institute for Microelectronics and Microsystems (IMM-CNR), Viale F. S. d’Alcontres 31, Messina 98166, Italy; ∥ Istituto di Fotonica e Nanotecnologie, Consiglio Nazionale delle Ricerche, Piazza Leonardo da Vinci 32, Milano 20133, Italy

**Keywords:** SECARS, coherent Raman scattering, standoff spectroscopy, plasmonics, silicon nanowires

## Abstract

Surface-enhanced coherent anti-Stokes Raman scattering (SECARS) has been widely explored for ultrasensitive vibrational spectroscopy, primarily in microscopy configurations employing high-numerical-aperture optics. Here, we demonstrate standoff detection of molecular vibrational fingerprints using plasmon-enhanced time-resolved SECARS at a working distance of 20 cm. We employ Ag-decorated silicon nanowires as a plasmonic substrate supporting the analyte molecules. Broadband pump (575 nm) and Stokes (625 nm) femtosecond pulses together with a narrowband delayed probe (515 nm) are used to generate the SECARS signal, with excitation and anti-Stokes wavelengths overlapping the plasmonic response of the substrate. System performance is first calibrated through Raman and CARS measurements of liquid pyridine and compressed Na_2_DPA powder. SECARS spectra of Rhodamine B and Na_2_DPA deposited on Ag-SiNWs are then recorded in backscattering at 20 cm distance, revealing clear vibrational fingerprints down to 10^–7^ M for Rhodamine B and 10^–6^ M for Na_2_DPA. The Na_2_DPA measurements provide an estimate of the overall SECARS/Raman signal amplification, yielding an effective enhancement of the order of 10^13^ under the present experimental conditions. These results establish a proof-of-concept for plasmon-enhanced standoff vibrational spectroscopy from analyte-loaded nanostructured substrates, including dipicolinate targets relevant to bacterial-spore detection.

## Graphical abstract

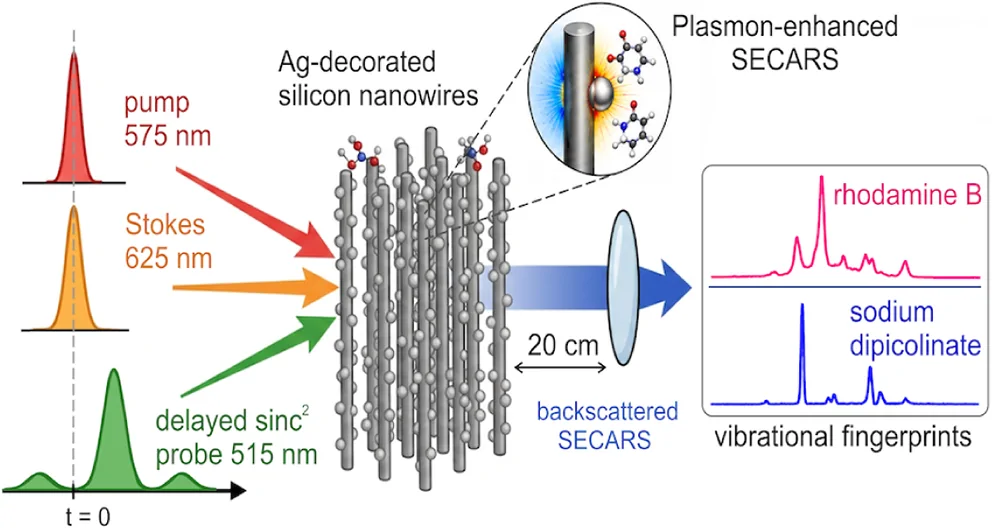

## Introduction

Vibrational spectroscopy provides a powerful route for identifying molecular species through their characteristic Raman fingerprints. Raman spectroscopy is widely used in chemical sensing, environmental monitoring, and biomedical analysis thanks to its label-free nature and high chemical specificity. However, spontaneous Raman scattering is intrinsically weak, with differential cross-sections typically on the order of 10^–30^–10^–29^ cm^2^ per molecule.^1,2^ Consequently, Raman measurements often require long acquisition times or high laser powers, particularly when probing low analyte concentrations. These limitations become even more severe in remote or standoff sensing configurations, where the weak Raman signal must be detected after propagation over macroscopic distances and with limited collection efficiency.^3,4^ Improving the sensitivity of vibrational spectroscopy therefore remains a central challenge for remote chemical detection and trace molecule analysis.

Coherent Raman scattering techniques provide a route to stronger vibrational signals by exploiting nonlinear interactions between multiple laser fields. Among these approaches, coherent anti-Stokes Raman scattering (CARS) is widely used for nonlinear spectroscopy and microscopy.^5–7^ In CARS, pump and Stokes fields excite a coherent vibrational polarization that is probed by a third optical field, producing a blue-shifted anti-Stokes signal. Because the third-order nonlinear polarization is proportional to the product of the pump, Stokes, and probe fields, and because the emitted signal can scale quadratically with the number of molecules under coherent conditions, coherent Raman processes can produce signals significantly stronger than spontaneous Raman scattering under appropriate conditions.^8,9^ However, when only a small number of molecules contribute to the interaction or excitation intensities must remain moderate, the expected signal advantage of coherent Raman processes may be reduced.^10^

Plasmonic nanostructures offer an effective strategy to further increase vibrational signal strength by amplifying the local electromagnetic field near metallic nanostructures. Surface-enhanced Raman scattering (SERS) exploits localized surface plasmon resonances to produce Raman enhancement factors of several orders of magnitude,^1,2,11^ Because nonlinear optical polarization depends on higher powers of the electric field, plasmonic near-field enhancement can lead to even stronger amplification of coherent Raman signals. This principle underlies surface-enhanced coherent anti-Stokes Raman scattering (SECARS), which combines coherent Raman excitation with plasmonic field localization near metallic nanostructures.^12–16^ Various plasmonic architectures have been explored to realize SECARS, including nanoparticle aggregates, nanoantennas, and engineered plasmonic nanostructures.^16,17^ Nevertheless, most SECARS demonstrations rely on microscope configurations employing high-numerical-aperture objectives and short working distances.

Among plasmonic substrates for Raman enhancement, silicon nanowire arrays decorated with noble-metal nanoparticles have attracted significant attention. These systems provide large surface areas, efficient light trapping, and dense distributions of plasmonic hotspots that enhance Raman signals from adsorbed molecules. Silver-coated silicon nanowires have been demonstrated as efficient large-area SERS substrates^18–20^ with subsequent studies reporting good spatial uniformity and strong electromagnetic field localization in hybrid metal–nanowire systems.^21–28^ In addition to plasmonic enhancement, random silicon nanowire networks exhibit strong light trapping and complex multiple-scattering effects, including coherent backscattering of Raman radiation.^29^ Fractal nanowire arrays have also been shown to increase optical interaction lengths and light trapping efficiency,^30^ while metal-assisted chemical etching provides a versatile route to fabricate nanowire-based optical sensing platforms.^31^

Despite the progress achieved in plasmon-enhanced Raman spectroscopy, its extension to remote or standoff sensing remains largely unexplored. Most demonstrations of surface-enhanced coherent Raman processes rely on microscope-based geometries, whereas remote vibrational detection using spontaneous or coherent Raman techniques remains challenging because of weak signal levels and limited collection efficiency in macroscopic geometries.^3,4,6,32^ Bridging the gap between plasmon-enhanced nonlinear spectroscopy and macroscopic-distance optical interrogation therefore represents an important step toward future remote chemical-sensing schemes.

In this work, we demonstrate laboratory-scale standoff detection of molecular vibrational fingerprints using time-resolved SECARS at a working distance of 20 cm. The experiments employ silicon nanowire arrays decorated with silver nanoparticles (Ag-SiNWs) acting as a plasmonic substrate. Broadband femtosecond pump and Stokes pulses together with a delayed narrowband probe generate the SECARS signal, which is collected in backscattering geometry and detected using a spectrometer with an intensified CCD camera. The excitation wavelengths are chosen to overlap the plasmonic response of the Ag-decorated nanowire substrate, maximizing nonlinear signal generation. By combining coherent Raman excitation with plasmonic enhancement provided by Ag-decorated silicon nanowires, we demonstrate standoff SECARS detection with an effective overall SECARS/Raman signal amplification of the order of 10^13^ for Na_2_DPA (NaDPA) under the present experimental conditions.

## Experimental/Materials

### Optical Setup

The layout of the experimental setup used for standoff time-resolved SECARS measurements is shown in Figure 1a. The excitation source starts with a femtosecond ytterbium laser system operating at a repetition rate of 1 kHz and delivering ∼250 fs pulses centered at 1030 nm. The output beam is divided into two branches used for generation of the broadband pump/Stokes pulses and of the probe pulse, respectively. A detailed description of the laser architecture will be reported in a dedicated publication.

**Figure 1:**
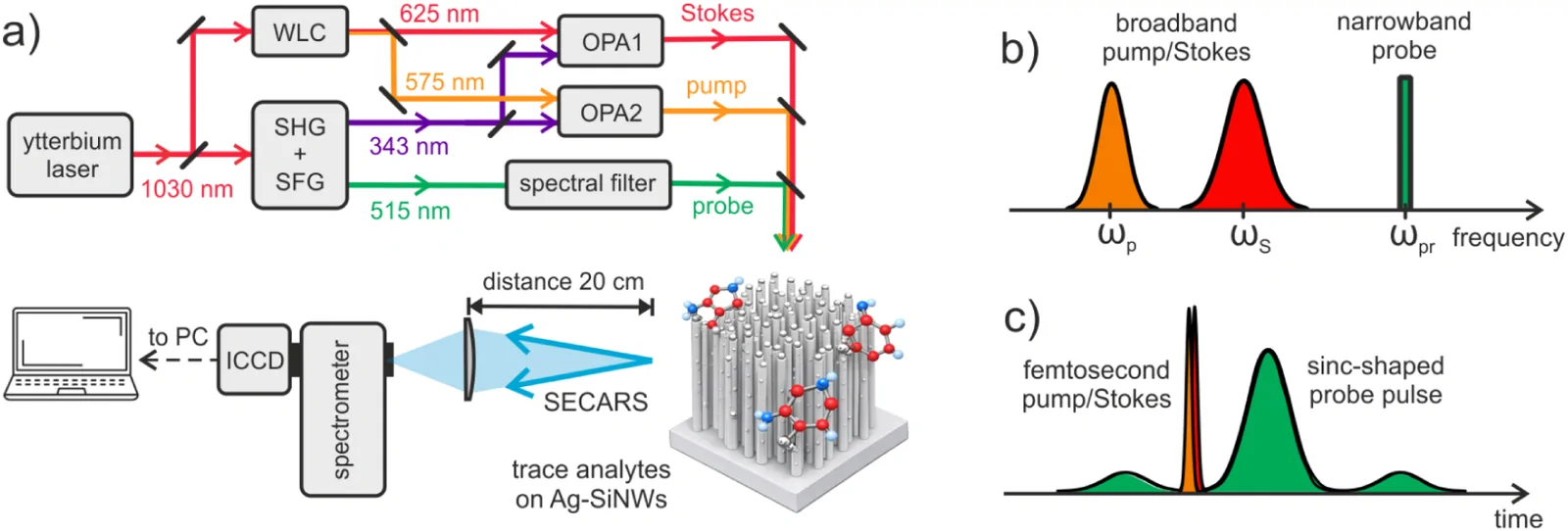
Experimental layout of the standoff time-resolved SECARS setup. (a) Laser source and nonlinear frequency conversion scheme: a Yb-based femtosecond laser at 1030 nm feeds a white-light continuum (WLC) and optical parametric amplifiers (OPA1, OPA2) to generate broadband pump (575 nm) and Stokes (625 nm) pulses, while a spectrally filtered second-harmonic provides a narrowband probe at 515 nm. (b) Spectral representation of the broadband pump and Stokes fields and of the narrowband probe in the frequency domain. (c) Temporal pulse sequence showing femtosecond pump/Stokes excitation and delayed narrowband probe for time-resolved detection. The beams are focused onto Ag-decorated silicon nanowires (Ag-SiNWs) at a working distance of 20 cm, and the backscattered anti-Stokes signal is collected and analyzed by a spectrometer equipped with an intensified CCD camera.

In the first branch, a small fraction of the fundamental radiation is focused into a nonlinear medium to generate a white-light continuum (WLC), which acts as a broadband seed for optical parametric amplification. In the second branch the fundamental beam is frequency-doubled to produce the second harmonic at 515 nm. The 1030 and 515 nm beams are subsequently mixed through sum-frequency generation to produce ultraviolet third-harmonic pulses at 343 nm. The resulting 343 nm radiation is split into two beams that pump two independent optical parametric amplifiers (OPAs), both seeded by the same WLC. One OPA generates the pump pulses centered at 575 nm, while the second OPA produces the Stokes pulses centered at 625 nm.

At the sample, the pump pulses at 575 nm have a spectral bandwidth of approximately 16 nm (fwhm, 480 cm^–1^), corresponding to a temporal duration of about 41 fs, and an average power of ∼2.5 mW. The Stokes pulses at 625 nm have a spectral bandwidth of about 28 nm (fwhm, 720 cm^–1^), a temporal duration of ∼30 fs, and an average power of ∼1 mW. The broad bandwidths of the pump and Stokes pulses enable simultaneous excitation of a wide range of Raman frequencies within the molecular fingerprint region.

The probe pulse is derived from the residual 515 nm radiation remaining after the sum-frequency generation stage. This beam is spectrally filtered by a zero-dispersion pulse shaper operating in the Fourier plane. A narrow slit placed in the Fourier plane imposes an approximately rectangular spectral profile (see Figure 1b) with a bandwidth of about 10 cm^–1^. This spectral shaping produces a probe pulse with a temporal intensity profile proportional to *sinc* ^2^ and a duration of approximately 2 ps. After spectral filtering the probe beam has an average power of about 7 mW.

The *sinc* ^2^ temporal profile of the probe pulse plays a key role in suppressing the nonresonant background in the CARS process.^33,34^ By adjusting the temporal delay between the probe pulse and the pump/Stokes pair, the excitation pulses can be positioned close to the first intensity zero of the probe temporal profile, where the instantaneous probe intensity vanishes (Figure 1c). Under these conditions the nonresonant four-wave-mixing contribution, corresponding to a simultaneous interaction with the pump, Stokes and probe fields, is strongly suppressed, while the vibrational coherence excited by the pump and Stokes pulses is still efficiently probed.

The pump, Stokes and probe beams are spatially combined using dichroic mirrors and collinearly focused onto the sample, as illustrated in Figure 1a. For the experiments reported here the beam diameter on the target is approximately 100 μm. The sample is positioned at a distance of about 20 cm from the focusing optics in a backscattering geometry.

The anti-Stokes radiation generated in the sample is collected by a 2-in. lens and directed to an imaging spectrometer equipped with a microchannel plate (MCP) intensified CCD detector (iCCD). Optical filters are used to reject the excitation beams and isolate the anti-Stokes signal, allowing detection of the vibrational fingerprint of the molecules interacting with the plasmonic substrate. Additional experimental details are provided in the Supporting Information.

### Fabrication and Characterization of Ag-SiNW Substrates

The plasmonic substrates employed in the SECARS experiments consist of Ag-SiNWs. The nanowire arrays were fabricated by metal-assisted chemical etching (MACE) of crystalline silicon wafers, which provides a scalable route to obtain dense forests of vertically aligned nanowires with extremely high surface area.

The fabrication process starts from a silicon wafer coated with a thin gold catalytic layer deposited by electron-beam evaporation. During the MACE process, performed in an aqueous solution containing HF and H_2_O_2_, the Au layer acts as a catalytic template at the Au/Si interface. The difference in electronegativity between Au and Si drives the local oxidation of silicon, while the resulting oxide is continuously dissolved by HF. This process leads to the formation of a dense vertical array of silicon nanowires with typical diameters of approximately 7 nm and lengths of about 3.5 μm, corresponding to a nanowire density on the order of 10^12^ cm^–2^.

To enable efficient attachment of metallic nanoparticles, the nanowire surfaces were chemically functionalized using alkoxysilane molecules. Three different surface treatments were investigated: (3-aminopropyl)­triethoxysilane (APTES), (3-mercaptopropyl)­trimethoxysilane (MPTMS), and (3-glycidyloxypropyl)­trimethoxysilane (GPTMS). These molecules introduce different functional groups on the nanowire surface that promote binding of silver nanoparticles from a colloidal suspension. Silver nanoparticles were synthesized through a citrate reduction method and subsequently immobilized on the functionalized nanowire surfaces by immersion of the substrates in the nanoparticle colloidal solution. Scanning electron microscopy (SEM) revealed that APTES functionalization provides the most uniform and homogeneous coverage of Ag nanoparticles along the nanowire sidewalls, whereas bare SiNWs and the other functionalization routes result in significantly lower nanoparticle densities.

Figure 2a shows a representative cross-sectional SEM image of the optimized Ag-SiNWs substrate obtained using APTES functionalization. The image highlights the dense vertical nanowire forest and the presence of Ag nanoparticles distributed along the nanowire walls, creating numerous nanoscale junctions that can act as plasmonic hotspots for electromagnetic field localization. A statistical analysis of the nanoparticle size distribution was performed using multiple SEM images and image-processing analysis. The resulting histogram is reported in Figure 2b. The distribution is well described by a log-normal function, yielding an average particle radius of approximately 15 ± 3 nm. Nanoparticles in this size range are known to support localized surface plasmon resonances in the visible spectral region. The optical response of the plasmonic substrate was further characterized by diffuse reflectance measurements in the visible range. To isolate the optical contribution of the Ag nanoparticles, an apparent absorbance spectrum was derived from reflectance data by comparing the response of the Ag-decorated SiNWs with that of the pristine nanowire substrate. The resulting spectrum is shown in Figure 2c.

**Figure 2:**
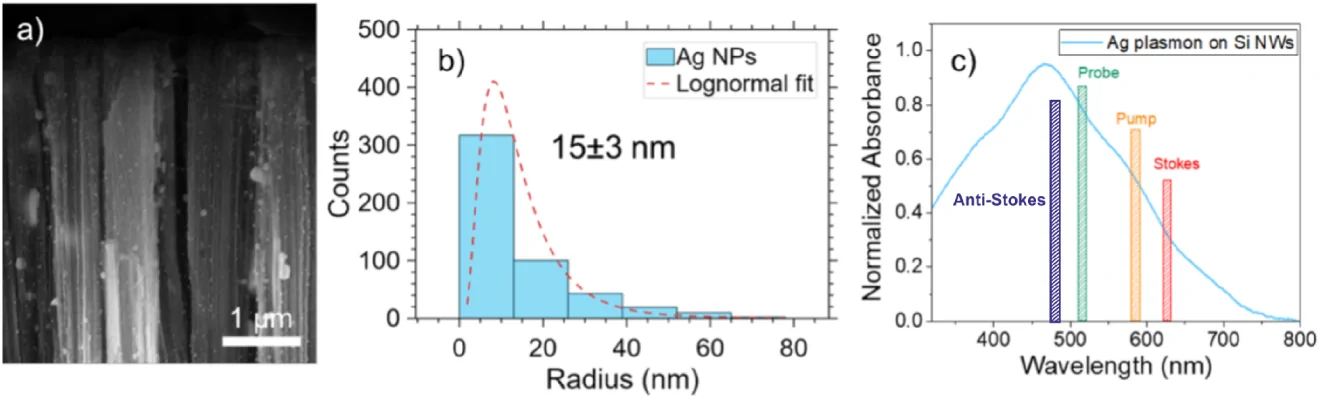
Morphological and optical characterization of the Ag-decorated silicon nanowire (Ag-SiNWs) substrate. (a) Cross-sectional scanning electron microscopy (SEM) image of the silicon nanowire array functionalized with APTES and decorated with Ag nanoparticles. The vertically aligned nanowires form a dense three-dimensional network with nanoparticles distributed along the nanowire sidewalls. (b) Statistical size distribution of the Ag nanoparticles extracted from SEM images. The histogram is fitted with a log-normal function, yielding an average particle radius of approximately 15 ± 3 nm. (c) Apparent absorbance spectrum derived from reflectance measurements by comparing the Ag-decorated SiNWs substrate with the pristine SiNW sample, highlighting the spectral region relevant to the SECARS excitation wavelengths. Vertical dashed lines indicate the wavelengths of the probe (515 nm), pump (575 nm), Stokes (625 nm), and the corresponding anti-Stokes emission (≃475–490 nm) for the Raman modes investigated in this work.

The analysis reveals a broad plasmonic feature extending across the visible range, with a maximum around 450–475 nm. This red-shift with respect to the typical plasmon resonance of colloidal Ag nanoparticles (around ∼400 nm) arises from the interaction between the nanoparticles and the silicon nanowire environment, as well as from electromagnetic coupling between neighboring particles. The excitation wavelengths used in the SECARS experiment lie within the broad plasmonic band of the Ag-SiNWs substrate: the probe (515 nm), pump (575 nm), and Stokes (625 nm) overlap the resonance, while the corresponding anti-Stokes emission (≃475–490 nm) lies close to its maximum. This spectral overlap ensures that the excitation fields experience significant local enhancement at the surface of the Ag nanoparticles, thereby favoring efficient nonlinear signal generation in the SECARS process. In addition to enhancing the excitation fields, the plasmonic resonance also spectrally overlaps with the anti-Stokes radiation generated in the SECARS process. For the Raman modes investigated in this work (around 1000–1600 cm^–1^), the corresponding anti-Stokes wavelengths fall in the ∼475–490 nm range, close to the maximum of the plasmonic response. This spectral overlap improves the radiative efficiency of the nonlinear signal generated at the plasmonic hotspots.

The combination of a three-dimensional nanowire architecture and uniform Ag nanoparticle decoration therefore provides a dense distribution of plasmonic hotspots capable of strongly enhancing the coherent Raman response of molecules adsorbed on the substrate surface.

## Results and Discussion

### System Validation and CARS/Raman Calibration

Prior to SECARS measurements on plasmonic substrates, the performance of the experimental system was validated using liquid pyridine as a reference sample. Pyridine is widely used as a calibration target in Raman spectroscopy because it exhibits strong and well-characterized vibrational modes in the molecular fingerprint region, in particular two intense ring breathing modes located near 1000 cm^–1^. These modes provide a convenient benchmark for evaluating spectral resolution and signal strength in coherent Raman experiments.

The reference measurement was performed using a cuvette containing liquid pyridine placed in the same backscattering configuration employed for the SECARS experiments. The cuvette thickness was 0.5 mm, and the sample was positioned at a distance of 20 cm from the collection optics. The wavenumber calibration procedure for CARS and SECARS spectra is described in the Supporting Information.

Figure 3 shows the CARS measurement obtained with the pump, Stokes, and probe beams spatially and temporally overlapped in the sample. Figure 3a reports the measured CARS spectrogram as a function of Raman shift and probe delay. As expected in time-resolved CARS measurements, a strong nonresonant four-wave-mixing background is observed when the pump, Stokes, and probe pulses temporally overlap. By increasing the probe delay, the nonresonant contribution rapidly decays and the resonant vibrational response of the sample becomes clearly visible.

**Figure 3:**
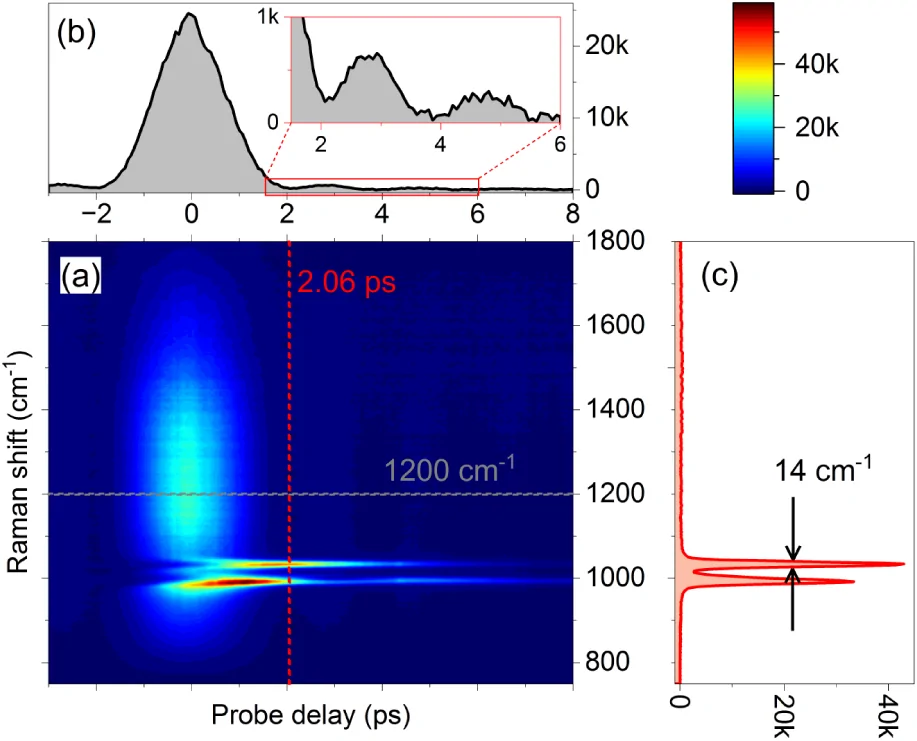
Standoff CARS spectrum of pure liquid pyridine. The sample, contained in a 0.5 mm cuvette, is positioned 20 cm from the collection lens. Average powers are 0.1 mW (pump), 0.2 mW (Stokes), and 0.1 mW (probe) at a repetition rate of 1 kHz. Integration time is 10 ms (10 laser shots). (a) CARS spectrogram as a function of Raman shift and probe delay. (b) Horizontal cross-section at 1200 cm^–1^ showing the temporal dependence of the CARS signal; the inset highlights the sinc^2^ intensity profile associated with the narrowband probe pulse. (c) Vertical cross-section at a probe delay of 2.06 ps, corresponding to the CARS spectrum of pyridine. At this delay, pump and Stokes pulses coincide with a zero of the probe sinc^2^ temporal profile, resulting in a strongly suppressed nonresonant background.

Two intense vibrational modes of pyridine are observed at 993 cm^–1^ and 1032 cm^–1^, in good agreement with the well-known Raman spectrum of pyridine. The horizontal cross-section of the spectrogram at 1200 cm^–1^, shown in Figure 3b, highlights the sinc^2^ temporal profile of the narrowband probe pulse. This behavior is more clearly visible in the inset of Figure 3b. From this profile, it is possible to identify, at a probe delay of 2.06 ps, a vertical region of the spectrogram where the nonresonant background is strongly suppressed. The vertical cross-section taken at this delay is reported in Figure 3c and corresponds to the broadband CARS spectrum of pyridine, where the vibrational lines at 993 cm^–1^ and 1032 cm^–1^ are clearly resolved. The measured full width at half-maximum (fwhm) of the CARS peaks is approximately 14 cm^–1^, consistent with the spectral resolution determined by the bandwidth of the narrowband probe pulse.

For comparison, the spontaneous Raman spectrum of pyridine was measured using the same optical configuration by blocking the pump and Stokes beams and exciting the sample only with the probe beam at 515 nm. The result is shown in Figure 4. In this case, the probe beam had an average power of 0.1 mW at 1 kHz repetition rate, as in the CARS measurement. Because spontaneous Raman scattering is several orders of magnitude weaker than the coherent CARS signal, a much longer integration time of 1000 s (10^6^ laser shots) was required.

**Figure 4:**
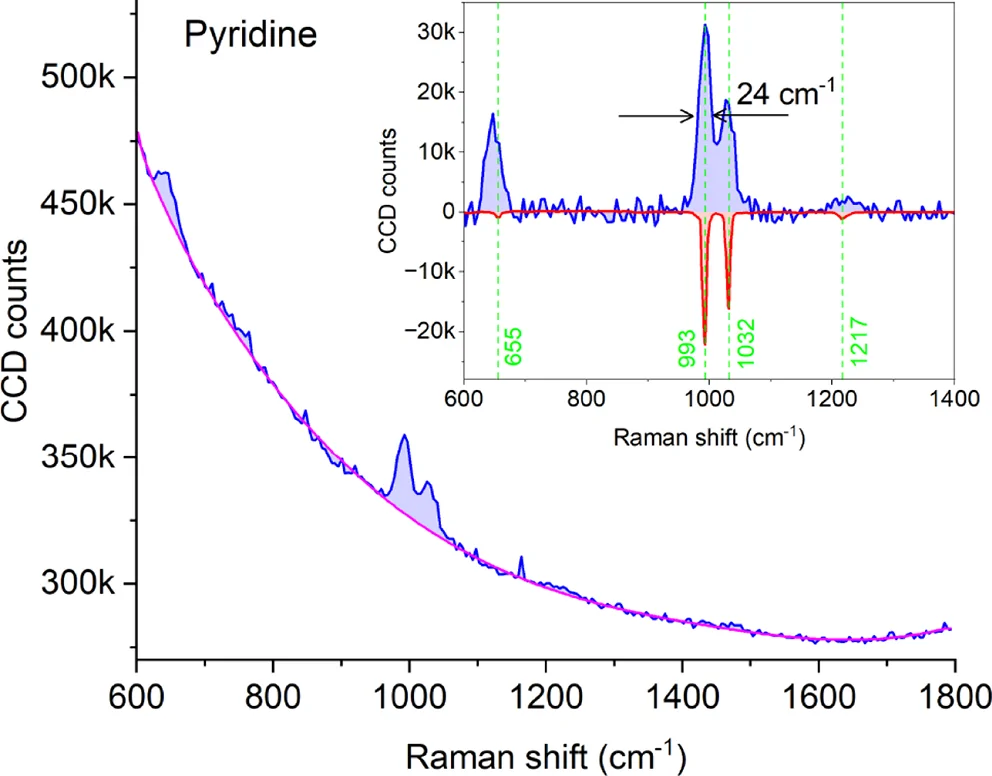
Spontaneous Stokes Raman spectrum of pure liquid pyridine measured in the same standoff configuration as in Figure 3, with pump and Stokes beams blocked. The probe average power is 0.1 mW at a repetition rate of 1 kHz. The total acquisition time is 1000 s (10^6^ laser shots). The blue curve shows the raw spectrum acquired with the intensified camera spectrometer, while the magenta curve represents the fitted baseline. Inset: Baseline-corrected experimental spectrum (blue) compared with the reference spontaneous Raman spectrum (red) of pyridine.^35^

The blue curve in Figure 4 shows the raw spectrum recorded by the intensified spectrometer, while the magenta curve represents the baseline obtained through a fitting procedure. The difference between the raw spectrum and the fitted baseline is reported in the inset of Figure 4 (blue curve), together with a reference spontaneous Raman spectrum of pyridine measured using a state-of-the-art commercial Raman spectrometer (red curve) taken from ref 35. The spectra reveal the main pyridine Raman bands at 993 and 1032 cm^–1^, together with weaker features at 655 and 1217 cm^–1^. The observed peak positions are in good agreement with the reference spontaneous Raman spectrum, confirming the correct identification of the vibrational modes detected in the experiment. The same pyridine vibrational modes observed in the CARS measurements are clearly visible in the Raman spectrum. In particular, the peak at 993 cm^–1^ appears with a measured width of approximately 24 cm^–1^. This broader line width compared to the CARS measurement is due to the different spectrometer settings used for the Raman acquisition. In order to increase the detected spontaneous Raman signal, the entrance slit of the spectrometer was opened to twice the width used in the CARS measurements, effectively doubling the spectral bandwidth admitted by the spectrometer and reducing the spectral resolution.

The comparison between the coherent CARS signal shown in Figure 3 and the spontaneous Raman signal reported in Figure 4 allows a quantitative estimate of the effective CARS/Raman enhancement under the present experimental conditions. The two signals were corrected for the different acquisition times, spectrometer slit widths, detector quantum efficiencies, and MCP gain settings, as described in the Supporting Information. This procedure gives $EF_{\mathrm{CARS}/\mathrm{Raman}}^{\mathrm{pyridine}}≃9.6\times 10^{5}$ . Thus, for the 993 cm^–1^ pyridine mode, the CARS process provides nearly 6 orders of magnitude more detected signal than spontaneous Raman scattering under the present measurement conditions. This result validates the coherent Raman performance of the setup prior to the SECARS measurements on plasmonic substrates.

### CARS/Raman Comparison on Compressed NaDPA Powder

To address the dependence of the CARS/Raman enhancement on the physical state and optical geometry of the sample, we performed an additional comparison between spontaneous Raman and CARS signals using compressed NaDPA powder. This measurement provides a more relevant reference for powder-like or surface-deposited samples than the liquid-pyridine calibration, because in strongly scattering solids the effective interaction length is limited by the penetration depth of the excitation fields rather than by the full optical path length of a cuvette. NaDPA was selected because dipicolinate salts are important markers for bacterial endospores. Bacillus spores contain calcium dipicolinate, CaDPA, which accounts for approximately 15–20% of the dry spore mass,^36^ depending on the species. The Raman spectra of bacterial endospores are therefore dominated by vibrational modes of CaDPA, including characteristic bands near 659, 824, 1017, 1395, 1446, and 1572 cm^–1^. Sodium dipicolinate provides a convenient substitute for CaDPA, with a closely related Raman fingerprint and easier preparation.

Figure 5 compares the reference micro-Raman spectrum of NaDPA powder with the spontaneous Raman and CARS spectra acquired in the present standoff configuration. The standoff spontaneous Raman spectrum was acquired using the same geometry employed for the pyridine Raman measurement, but with a total integration time of 10 000 s, obtained by averaging ten consecutive 1000-s acquisitions, and with an increased probe power of 1 mW at 515 nm. These conditions were required because the Raman signal from the compressed powder is substantially weaker than that from liquid pyridine, mainly due to the reduced optical penetration depth in the strongly scattering solid.

**Figure 5:**
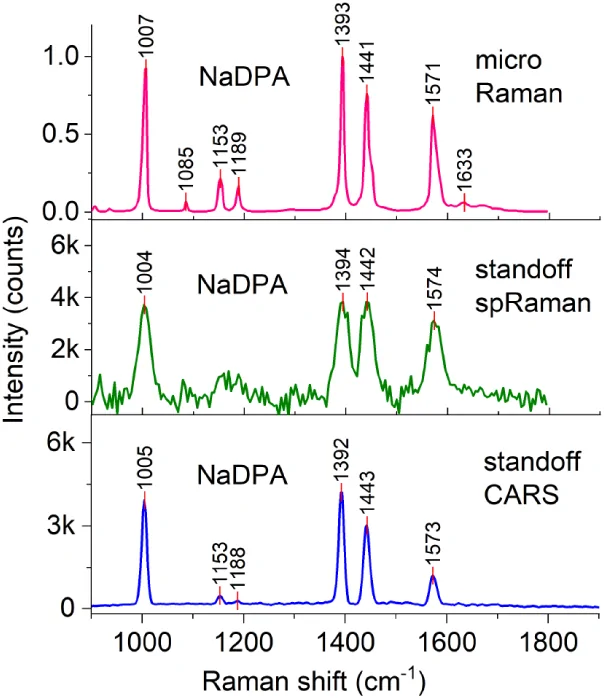
Comparison between spontaneous Raman and CARS spectra of NaDPA powder. Top: reference micro-Raman spectrum of NaDPA powder.^37^ Middle: spontaneous Raman spectrum measured in the current standoff configuration using only the 515 nm probe beam, with an average power of 1 mW. The spectrum is the average of ten consecutive acquisitions with 1000 s integration time each. Bottom: standoff CARS spectrum acquired from compressed NaDPA powder with pump, Stokes, and probe beams incident on the sample. Average powers are 0.5 mW (pump), 0.5 mW (Stokes), and 1 mW (probe); integration time is 100 ms (100 laser shots).

The CARS spectrum was acquired by overlapping the pump, Stokes, and probe beams on the compressed NaDPA sample. The integration time was 100 ms, and the average powers were 0.5 mW, 0.5 mW, and 1 mW for the pump, Stokes, and probe beams, respectively. The main Raman bands of NaDPA are clearly reproduced in the CARS spectrum, confirming that broadband time-resolved coherent Raman detection can be extended to powder-like targets relevant to standoff sensing.

This comparison also highlights an important difference between liquid and powder samples. In the liquid-pyridine cuvette, the number of molecules contributing to the CARS polarization is proportional to the optical path length of the cuvette, which is typically in the 0.1–1 mm range. In contrast, in a compressed powder, the effective interaction length is limited by the penetration depth of the excitation fields in a highly scattering medium, which is of the order of 10 μm.^38^ This reduction has a moderate effect on spontaneous Raman scattering, which scales linearly with the number of interrogated molecules, but it has a much stronger impact on CARS under coherent buildup conditions, where the generated signal can scale quadratically with the number of coherently contributing molecules. Therefore, the CARS/Raman enhancement measured in liquid pyridine represents an upper-bound calibration of the coherent Raman advantage in an optically homogeneous medium, whereas the NaDPA powder measurement provides a more appropriate reference for solid and surface-deposited analytes.

The spontaneous Raman and CARS spectra of compressed NaDPA powder were used to estimate an effective CARS/Raman enhancement for a strongly scattering solid analyte. After correction for acquisition time, excitation powers, spectrometer slit width, detector quantum efficiency, and MCP gain, as detailed in the Supporting Information, we obtain $EF_{\mathrm{CARS}/\mathrm{Raman}}^{\mathrm{NaDPA}}≃8\times 10^{4}$ for the NaDPA band near 1005 cm^–1^. This value should be interpreted as an effective experimental signal gain under the present measurement conditions, rather than as an intrinsic molecule-independent enhancement factor.

### Plasmon-Enhanced SECARS Detection on Ag-SiNWs

#### SECARS Detection of Rhodamine B as a Benchmark Dye

After the preliminary characterization performed on pyridine, we moved to Rhodamine B (RhB) as a molecular target. Rhodamine dyes are widely used benchmark molecules in Raman and SERS because they exhibit a rich vibrational fingerprint in the 1000–1700 cm^–1^ spectral region. In contrast to pyridine, which is characterized by only a few strong vibrational lines, RhB presents many Raman-active modes distributed across a broad spectral range. This makes it a particularly suitable test molecule to evaluate the spectral bandwidth and resolving capability of the SECARS system developed in this work.

It should be noted that Rhodamine B is investigated under partially resonant conditions in the present experiment. RhB exhibits a broad electronic absorption band in the visible range, extending approximately from 500 to 580 nm and peaked around 555 nm. This band overlaps with the probe wavelength at 515 nm and partly with the pump wavelength at 575 nm. Therefore, the CARS and SECARS signals of RhB measured here contain a resonance-enhanced contribution to the nonlinear vibrational response.

The experiments were carried out using the same excitation configuration described above, with pump, Stokes, and probe wavelengths of 575, 625, and 515 nm, respectively. RhB solutions were prepared in isopropanol at different molar concentrations and drop-cast onto the Ag-SiNW substrates. After solvent evaporation, the molecules remained adsorbed on the plasmonic nanostructured surface. The backscattered anti-Stokes radiation generated through the SECARS process was collected in reflection at a distance of 20 cm and analyzed by the spectrometer.

Before analyzing the concentration-dependent response, we first verified the suppression of the nonresonant background by the delayed-probe detection scheme. Figure 6 compares SECARS spectra of RhB acquired at zero pump–Stokes/probe delay and at a probe delay of 2.06 ps, under otherwise identical experimental conditions. At zero delay, the pump, Stokes, and probe fields temporally overlap at the sample, so that the detected signal is dominated by the instantaneous nonresonant four-wave-mixing response of the substrate and molecular environment. The resonant vibrational contribution is then superimposed on the nonresonant background, and interference between the two terms can modify the apparent peak amplitudes and produce asymmetric or dispersive lineshapes, making the extraction of the resonant molecular fingerprint less straightforward. When the probe pulse is delayed to 2.06 ps, the pump and Stokes pulses coincide with a zero of the sinc^2^ temporal profile of the narrowband probe. Under these conditions, the instantaneous nonresonant contribution is strongly reduced, while the vibrational coherence excited by the pump and Stokes pulses can still be probed. As a result, the delayed SECARS spectrum shows a much clearer molecular fingerprint of RhB, with well-defined vibrational bands across the 1000–1700 cm^–1^ region.

**Figure 6:**
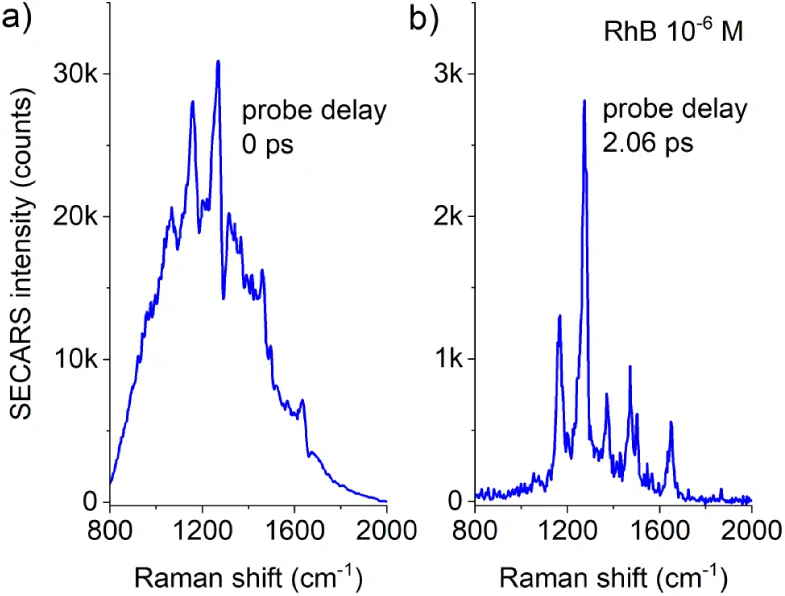
Effect of time-resolved detection on the suppression of the nonresonant background in SECARS measurements of Rhodamine B deposited on Ag-SiNWs. The backscattered signal is collected by a lens placed 20 cm from the sample. Pump (575 nm), Stokes (625 nm), and probe (515 nm) beams have average powers of 20 μW, 40 μW, and 10 μW, respectively, at a repetition rate of 1 kHz. Integration time is 10 ms (10 laser shots). Pump and Stokes pulses are delayed by (a) 0 ps and (b) 2.06 ps with respect to the probe pulse. At zero delay, the spectrum is dominated by the nonresonant four-wave-mixing background, whereas at 2.06 ps the nonresonant contribution is strongly suppressed and the resonant vibrational fingerprint of Rhodamine B becomes clearly visible.

This comparison also illustrates an important aspect of time-resolved CARS/SECARS detection. Delayed probing efficiently suppresses the instantaneous nonresonant background, but the measured spectrum can, in principle, be influenced by the vibrational dephasing times and Raman strengths of the individual modes. Short-lived coherences, typically associated with broader Raman bands, may be attenuated at picosecond probe delays. Weak Raman modes can also become less visible after delayed probing because their resonant contribution is intrinsically smaller and competes less favorably with residual background and noise. This behavior is observed in liquid pyridine, where broad or weak components become almost invisible at delays of about 2 ps. In the present RhB measurements, however, the main vibrational fingerprint remains clearly visible at a delay of 2.06 ps, as confirmed by comparison with the spontaneous micro-Raman reference spectrum in Figure 8. This is likely due to the presence of several intense and relatively well-defined Raman bands, together with the partially resonant character of RhB under the present excitation conditions.

Figure 7a reports the SECARS spectra measured for RhB at different molar concentrations. The figure also includes the SECARS spectrum of the bare Ag-SiNWs substrate, acquired under the same experimental conditions but without deposited RhB. As the concentration decreases, the overall signal intensity is progressively reduced, while the spectral positions of the vibrational bands remain unchanged, further supporting the molecular origin of the detected signal. Even at the lowest investigated concentrations, the main Raman bands remain distinguishable from the background. This observation demonstrates that the combination of coherent Raman excitation and plasmonic enhancement provided by the Ag-SiNWs substrate yields sufficient signal strength to enable reliable detection. Several vibrational modes characteristic of RhB are simultaneously observed across the molecular fingerprint region. In particular, distinct features appear between approximately 1000 and 1700 cm^–1^, indicating that the broadband excitation scheme allows multiple Raman-active modes to be probed in a single measurement.

**Figure 7:**
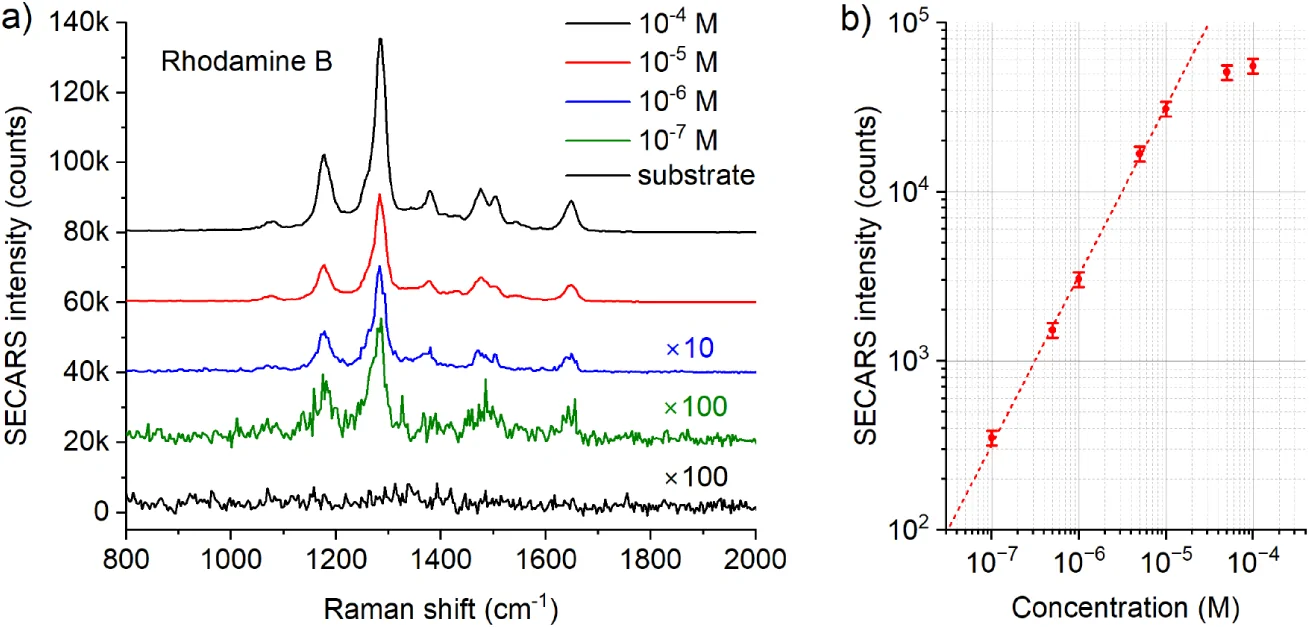
Concentration-dependent standoff SECARS detection of Rhodamine B deposited on Ag-SiNWs. Experimental conditions are the same as in Figure 6b. (a) Background-subtracted SECARS spectra acquired at different molar concentrations and vertically offset for clarity. (b) SECARS intensity of the Raman peak at 1282.6 cm^–1^ as a function of molar concentration. Each data point represents the average of 10 measurements acquired at different sample spots by vertical translation of the substrate, orthogonal to the laser propagation direction. Error bars indicate ±1 standard deviation for each concentration. The red dashed line represents a linear fit of the data up to a molar concentration of 10^–5^ M.

The concentration dependence was quantified from the intensity of the 1282.6 cm^–1^ band, as shown in Figure 7b. The signal follows an approximately linear trend up to 10^–5^ M and deviates from linearity at higher concentrations. The linear regime is consistent with a response dominated by molecules coupled to spatially separated plasmonic hotspots, whose emissions add predominantly incoherently. The sublinear behavior at higher concentrations is attributed to the increasing contribution of molecules located in lower-enhancement regions, aggregates, multilayers, or farther from the metal surface. A more detailed discussion is provided in the Supporting Information.

Figure 8 compares the SECARS spectrum of RhB on Ag-SiNWs with the spontaneous Raman spectrum of pure RhB powder and with a time-delayed CARS spectrum of compressed RhB powder. The delayed CARS spectrum reproduces the main Raman bands without major spectral shifts, whereas larger shifts, changes in relative intensities, and residual asymmetries are observed in the SECARS spectrum. These differences are attributed mainly to molecule–substrate interactions, local molecular orientation, mode-dependent plasmonic enhancement, and the partially resonant character of RhB under the present excitation conditions. A more detailed discussion of the spectral comparison is provided in the Supporting Information.

**Figure 8:**
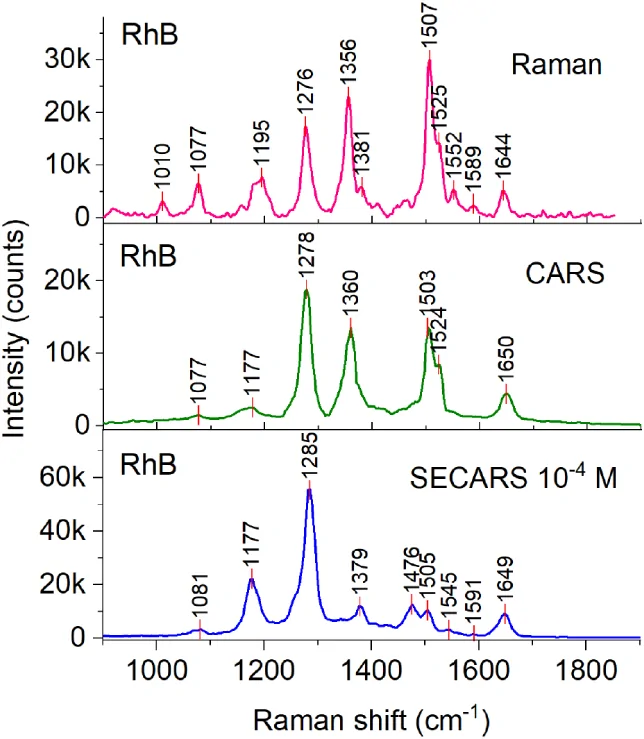
Comparison between spontaneous Raman, time-delayed CARS, and SECARS spectra of Rhodamine B. Top: spontaneous Raman spectrum of pure Rhodamine B powder measured with a commercial micro-Raman spectrometer.^39^ Middle: time-delayed CARS spectrum of compressed Rhodamine B powder acquired at a probe delay of 2.06 ps. Bottom: SECARS spectrum of Rhodamine B deposited on Ag-SiNWs at a concentration of 10^–4^ M, acquired under the same delayed-probe conditions as in Figure 7.

Despite these differences, the main spectral features remain clearly attributable to RhB, demonstrating that the Ag-SiNWs substrate preserves the molecular fingerprint sufficiently well for chemical identification while providing strong plasmonic amplification of the coherent Raman response. The spatial reproducibility of the SECARS response across the Ag-SiNWs substrate was also evaluated and is reported in the Supporting Information.

To further quantify the performance of the plasmonic substrate, we estimated the effective SECARS/CARS enhancement for RhB by comparing the SECARS signal obtained on Ag-SiNWs with the delayed CARS signal measured from compressed RhB powder. The comparison was normalized to the integration time, excitation-power product, and estimated number of probed molecules, as detailed in the Supporting Information. This procedure gives $EF_{\mathrm{SECARS}/\mathrm{CARS}}^{\mathrm{RhB}}≃2\times 10^{7}$ .

Since this enhancement is obtained by comparing CARS and SECARS signals from the same molecule under the same pump, Stokes, and probe wavelengths, the molecular resonance contribution is expected to be present in both measurements and to partially cancel in the ratio. Nevertheless, this value should be interpreted as an effective substrate-induced enhancement under RhB resonant conditions, rather than as a molecule-independent plasmonic enhancement factor. This result confirms the strong electromagnetic amplification provided by the Ag-SiNW substrate and falls within the range of effective enhancement values reported for plasmon-enhanced Raman and coherent Raman processes in nanostructured metallic substrates.^1,6,16^

#### SECARS Detection of NaDPA as a Bacterial-Spore Marker

To further evaluate the applicability of the standoff SECARS approach to analytes relevant to biological threat detection, we performed additional measurements on NaDPA deposited on Ag-SiNWs. NaDPA was selected as a convenient water-soluble form of dipicolinate, which is closely related to calcium dipicolinate, a major molecular marker of bacterial endospores.^36,40^ In aqueous solution, sodium acts mainly as a solvated counterion, while the vibrational fingerprint is expected to originate from the dipicolinate anion adsorbed in the vicinity of the Ag nanostructured surface.

NaDPA aqueous solutions were prepared at different molar concentrations and deposited onto the Ag-SiNWs substrate using calibrated 1 μL droplets, following the same drop-casting procedure used for RhB. After solvent evaporation, the dried residue was probed in the standoff SECARS configuration. The excitation powers were the same as those used for the RhB SECARS measurements, namely 20 μW, 40 μW, and 10 μW for the pump, Stokes, and probe beams, respectively. Because the NaDPA SECARS signal was weaker than that of RhB, the integration time was increased to 1 s.

Figure 9a shows the SECARS spectra obtained for different NaDPA concentrations. The main vibrational bands of dipicolinate are clearly observed, including the intense ring-breathing mode near 1007 cm^–1^ and additional bands in the 1300–1600 cm^–1^ region, associated with pyridine-ring vibrations and carboxylate-related modes. These bands are consistent with the characteristic Raman fingerprint of dipicolinate salts and confirm that the detected signal originates from the NaDPA analyte deposited on the plasmonic substrate.

**Figure 9:**
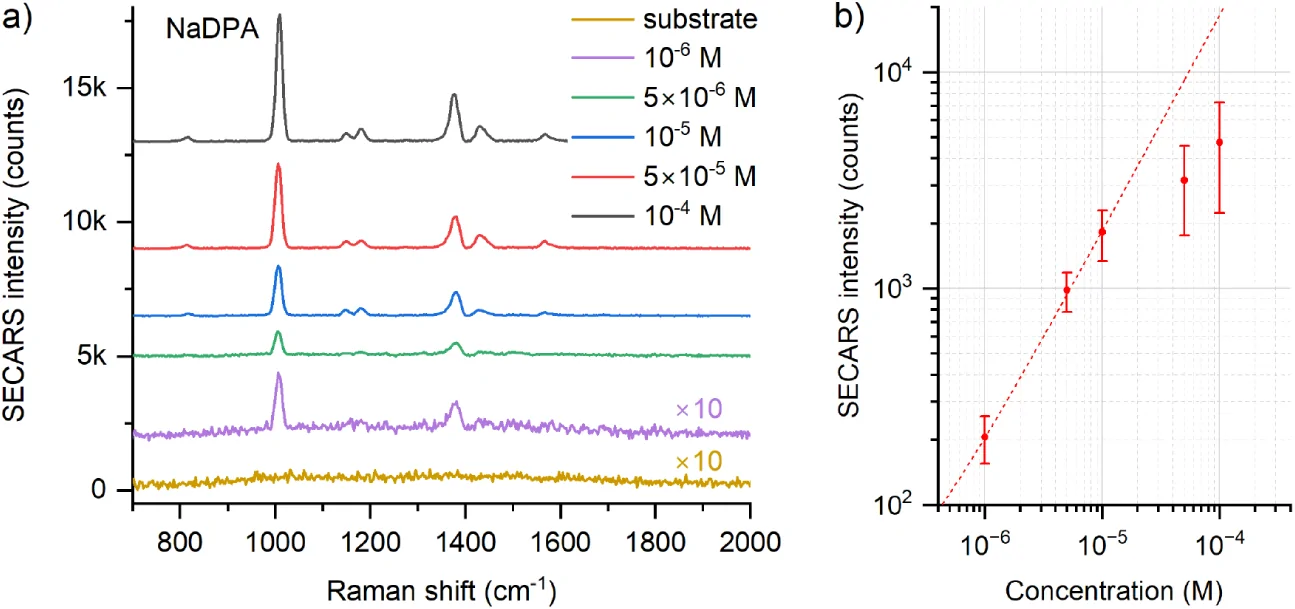
Standoff SECARS detection of NaDPA deposited on Ag-SiNWs. Experimental conditions are the same as in Figure 7, except for the integration time of 1 s (1000 laser shots). (a) Background-subtracted SECARS spectra of NaDPA at different molar concentrations and vertically offset for clarity. (b) SECARS intensity of the Raman peak at 1007 cm^–1^ as a function of molar concentration. Each data point represents the average of 10 measurements acquired at different sample spots by vertical translation of the substrate, orthogonal to the laser propagation direction. Error bars indicate ±1 standard deviation for each concentration. The red dashed line represents a linear fit of the data up to a molar concentration of 10^–5^ M.

As in the case of RhB, the SECARS spectrum can differ from the corresponding spontaneous Raman or bulk CARS spectrum in terms of relative peak intensities and, in some cases, peak positions. In particular, the interaction of the dipicolinate anion with Ag nanoparticles, local molecular orientation, and the heterogeneous distribution of electromagnetic hotspots can selectively enhance specific vibrational modes. The modes involving the pyridine ring and the carboxylate groups are expected to be particularly sensitive to adsorption geometry and local chemical environment.^41^

The concentration dependence of the NaDPA SECARS signal was quantified by plotting the intensity of the 1007 cm^–1^ band as a function of concentration, as shown in Figure 9b. The signal increases approximately linearly up to about 10^–5^ M, while deviations from linearity are observed at higher concentrations. In contrast to RhB, NaDPA does not exhibit a strong visible fluorescence background under the present excitation conditions. The deviation is therefore attributed mainly to drying-induced redistribution of the salt deposit, local aggregation or crystallization, and a decreasing fraction of molecules effectively coupled to the most active plasmonic hotspots. A more detailed discussion is provided in the Supporting Information.

To further assess the spectral assignment, Figure 10 compares the SECARS spectrum of NaDPA on Ag-SiNWs with a spontaneous Raman spectrum of dipicolinic acid (DPA) taken from ref 42. The main DPA/dipicolinate fingerprint is preserved, in particular the intense band near 1007 cm^–1^ associated with the pyridine-ring breathing mode. Differences in relative intensities and peak positions are expected for surface-enhanced measurements on silver and are attributed to adsorption geometry, molecule–surface interactions, and mode-dependent plasmonic enhancement. A more detailed assignment of the observed bands is provided in the Supporting Information.

**Figure 10:**
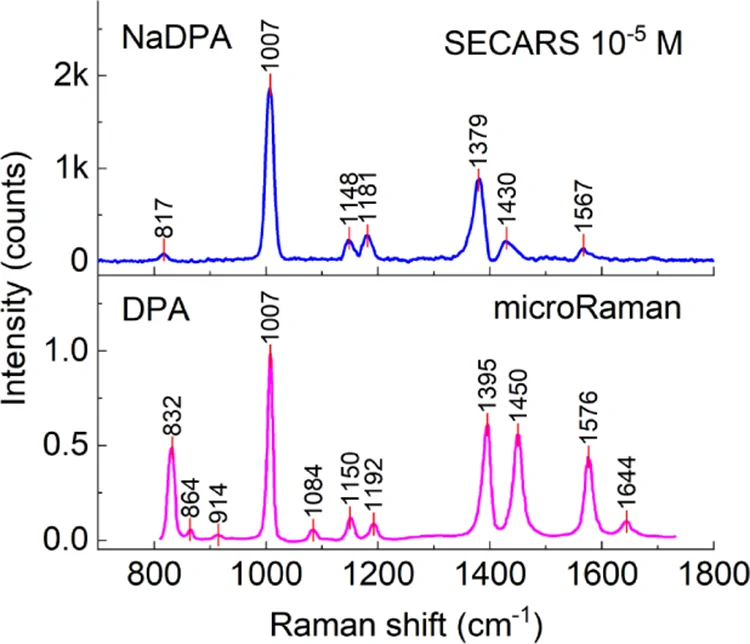
Comparison between the spontaneous Raman spectrum of dipicolinic acid (DPA) and the standoff SECARS spectrum of NaDPA deposited on Ag-SiNWs. Top: SECARS spectrum of NaDPA deposited on Ag-SiNWs at a concentration of 10^–5^ M, acquired under the same experimental conditions as in Figure 9. Bottom: spontaneous Raman spectrum of DPA, taken from ref 42.

The effective SECARS/CARS enhancement for NaDPA was estimated by comparing the SECARS signal at 10^–5^ M with the reference CARS signal measured from compressed NaDPA powder. After normalization to integration time, excitation-power product, and estimated number of probed molecules, as detailed in the Supporting Information, we obtain $EF_{\mathrm{SECARS}/\mathrm{CARS}}^{\mathrm{NaDPA}}≃1.4\times 10^{8}$ . This value confirms that the Ag-SiNWs substrate provides strong plasmonic amplification of the coherent Raman response also for dipicolinate analytes.

The measurements presented in this work provide a quantitative evaluation of the signal amplification achieved in the standoff time-resolved SECARS configuration. The enhancement factors reported here should be interpreted as effective experimental signal ratios under the stated measurement conditions, rather than as universal molecule-independent quantities.

The liquid-pyridine calibration gives $EF_{\mathrm{CARS}/\mathrm{Raman}}^{\mathrm{pyridine}}≃9.6\times 10^{5}$ , validating the coherent Raman performance of the setup. However, because this measurement benefits from coherent buildup over the cuvette path length, it should not be directly combined with SECARS/CARS enhancements measured on surface-deposited or powder-like analytes. A more internally consistent estimate is obtained from the NaDPA measurements, for which both the CARS/Raman and SECARS/CARS comparisons are performed on the same analyte. The product of $EF_{\mathrm{CARS}/\mathrm{Raman}}^{\mathrm{NaDPA}}≃8\times 10^{4}$ and $EF_{\mathrm{SECARS}/\mathrm{CARS}}^{\mathrm{NaDPA}}≃1.4\times 10^{8}$ gives an overall SECARS/Raman signal amplification of the order of 10^13^ under the assumptions and experimental conditions described above. The slightly larger effective SECARS/CARS enhancement estimated for NaDPA compared to RhB is consistent with the partial quenching of fluorescence and resonance-Raman contributions reported for Rhodamine dyes adsorbed on plasmonic nanoparticles.^43,44^ This suggests that the lower effective enhancement obtained for RhB may partly reflect its resonant dye character, whereas NaDPA behaves as a more nonresonant dipicolinate analyte under the present excitation conditions.

The enhancement observed in the present SECARS measurements arises from two distinct contributions: coherent Raman signal generation and local electromagnetic amplification at plasmonic hotspots. In addition, multiple scattering and light trapping in the Ag-decorated silicon nanowire network may increase the effective interaction length and redirect part of the generated anti-Stokes radiation toward the collection optics in backscattering geometry. This interpretation is consistent with previous studies showing enhanced optical trapping and coherent backscattering in random silicon nanowire networks^29,30^ as well as strong light–matter interaction in metal-decorated SiNW optical sensing platforms.^31^

A comparison with representative enhancement factors reported for SERS, CARS, and SECARS experiments is provided in Table S2 of the Supporting Information. Most reported SECARS demonstrations have been performed in microscopy configurations using high-numerical-aperture objectives, whereas standoff implementations remain comparatively rare. In this context, the present results show that coherent Raman excitation combined with Ag-SiNW plasmonic substrates can provide an effective SECARS/Raman amplification of the order of 10^13^ in a macroscopic backscattering geometry, as demonstrated by NaDPA.

The measurements were performed at a working distance of 20 cm and should be regarded as a laboratory proof-of-concept demonstration of standoff SECARS. In the present backscattering geometry, the detected signal is expected to scale approximately with the collection solid angle, and therefore as 1/*r* ^2^ for a fixed collection aperture. Extension to meter-scale distances would require compensation of the reduced collection efficiency by increasing the collection aperture, excitation pulse energies, integration time, or illuminated substrate area, while keeping the fluence below the damage threshold of the plasmonic substrate. A more detailed scaling estimate is reported in the Supporting Information. This distance scaling is consistent with our previous field-deployable standoff CARS implementation, where operation at a 1 m working distance was achieved by using higher pulse energies and a larger beam diameter on the target.^32^ This comparison supports the feasibility of extending the present SECARS geometry to meter-scale distances, provided that excitation energy, illuminated area, collection optics, and spectrometer coupling are optimized.

A further practical limitation of the present demonstration is that the analyte is deposited on the Ag-SiNWs substrate before optical interrogation. Therefore, the experiment should not be interpreted as direct standoff detection of an arbitrary contaminated surface without sample collection or transfer. Rather, it demonstrates remote optical interrogation of a plasmonic collection substrate. In practical sensing scenarios, such a geometry could be combined with sampling strategies in which analytes are first accumulated on a disposable or prepositioned plasmonic collector, for example, by passive deposition, aerosol collection, swab transfer, or contact sampling of contaminated surfaces. The SECARS measurement could then be performed remotely on the collector substrate, reducing the need for close optical access during the spectroscopic readout. Future development toward operational sensing would therefore require integration of the present optical approach with suitable sampling or preconcentration methods, in addition to the distance-scaling optimizations discussed above.

## Conclusions

Overall, these results demonstrate that plasmon-enhanced coherent Raman spectroscopy can enable vibrational fingerprint detection in standoff configurations. Even with relatively large illumination spots and limited collection efficiency, clear molecular signatures can be retrieved from trace quantities of analyte deposited on nanostructured plasmonic substrates. The additional NaDPA measurements further show that this approach can be extended from benchmark dye molecules to dipicolinate targets relevant to bacterial-spore detection, highlighting the potential of time-resolved SECARS as a sensitive readout method for analyte-loaded plasmonic substrates in future remote molecular-sensing schemes.

## Supplementary Material



## Data Availability

The data supporting the findings of this study are available from the corresponding author upon reasonable request.

## Notes

Experimental parameters of the standoff setup; pump and Stokes pulse characterization; NaDPA sample preparation; Raman, CARS, and SECARS spectral calibration; estimation of effective enhancement factors; concentration dependence and spectral assignments; spatial uniformity measurements; working-distance scaling analysis; and comparison with literature enhancement factors (PDF)
