# Supplementary material for: Standoff Time-Resolved SECARS on Ag-Decorated Silicon Nanowires
Source: ACS Photonics. 2026 Sep 24;13(19):5674–84. doi: 10.1021/acsphotonics.6c01317 (PMC13660374; doi:10.1021/acsphotonics.6c01317)
Supplement: Supplementary file 1 [file ph6c01317_si_001.pdf]

Supporting Information

Standoff Time-Resolved SECARS on Ag-Decorated Silicon Nanowires

Marco Polastri[1], Antonio Alessio Leonardi[2,3], Ileana Ielo[2], Barbara Fazio[3], Giulio Cerullo[1,4], Alessia Irrera[3], and Nicola Coluccelli*[1]

[1]Department of Physics, Politecnico di Milano, Piazza Leonardo da Vinci 32, 20132 Milano, Italy
[2]Department of Chemical, Biological, Pharmaceutical, and Environmental Sciences (ChiBioFarAm), University of Messina, Viale F. S. d'Alcontres 31, I-98166 Messina, Italy
[3]Institute for Microelectronics and Microsystems (IMM-CNR), Viale F. S. d'Alcontres 31, 98166 Messina, Italy
[4]Istituto di Fotonica e Nanotecnologie, Consiglio Nazionale delle Ricerche, Piazza Leonardo da Vinci 32, 20133 Milano, Italy

Contents: 11 pages, 2 figures, and 2 tables

Standoff setup experimental parameters

The excitation beams were focused onto the sample using a silver-coated metallic concave mirror with a diameter of 12.7 mm and a radius of curvature of 400 mm, corresponding to a focal length of 200 mm. Using the semi-aperture of the mirror, this corresponds to a focusing numerical aperture of approximately 0.032. The backscattered radiation was collected using a 1:1 imaging telescope composed of two lenses with focal length of 200 mm and diameter of 50.8 mm. The corresponding collection numerical aperture is approximately 0.127.

For a collection distance of 200 mm and a collection diameter of 50.8 mm, the collection half-angle is approximately 7.2°, giving a collection solid angle Ω of approximately 5.0×10^{-2} sr. For diffuse isotropic emission, this corresponds to a geometrical collection fraction $\Omega/4\pi$ of approximately 4.0×10^{-3}, i.e., about 0.4%.

The spectra were acquired with an Andor Kymera 328i spectrograph with an aperture of F/4.1 and focal length of 328 mm. A 1200 lines/mm grating was used, giving a spectral dispersion of 2.19 nm/mm and a spectral resolution better than 0.1 nm. Detection was performed with an Andor iStar intensified CCD camera equipped with a Gen3 18U-73 intensifier. The detector has a VIH photocathode covering the 280–910 nm spectral range, 16-bit digitization, and a minimum optical gate width below 2 ns. The camera was cooled to $-40\,^{\circ}$C during the measurements.

For CARS and SECARS measurements, the anti-Stokes signal was selected using an Omega Optical 500SP short-pass filter, with a cutoff wavelength of 500 nm, optical density larger than 5 in the rejection band, and transmission above 90% in the passband. For spontaneous Raman measurements, the elastic scattering was rejected using an EKSMA Optics 246-2506-514 notch filter. The rejection wavelength of this filter can be tuned by changing the angle of incidence; the filter provides optical density larger than 6 in the rejection band and transmission above 90% in the passband.

The beam quality of the pump and Stokes beams is mainly determined by the beam quality of the amplified optical-parametric-amplifier pump beam, namely the second harmonic at 515 nm generated from the 1030 nm fundamental. The latter has a beam-quality factor close to $M^2 \simeq 1.2$, and the pump and Stokes beams generated by the OPA are therefore expected to remain close to diffraction-limited propagation. The spot sizes at the sample position were determined according to the $d4\sigma$ beam-diameter definition [1].

For spontaneous Raman measurements and for backscattered CARS/SECARS measurements from powder or surface-deposited samples, the collected signal was treated as diffuse backscattered radiation. In these cases, the geometrical collection efficiency is first determined by the collection solid angle reported above. An additional throughput factor is introduced by the finite width of the entrance slit of the spectrometer.

The entrance slit was set to 100 μm for spontaneous Raman measurements and to 50 μm for CARS and SECARS measurements, as reported in the main text. Assuming an approximately circular top-hat image on the slit plane with an effective diameter of about 100 μm, the corresponding slit throughputs are approximately 1 for spontaneous Raman measurements and 0.61 for CARS/SECARS measurements.

*Corresponding author: nicola.coluccelli@polimi.it

For the liquid-pyridine CARS measurement in cuvette, the generated CARS field is coherent and directional. Therefore, unlike diffuse Raman or backscattered CARS/SECARS signals, its collection is not limited by the isotropic solid-angle factor. The dominant geometrical loss is instead associated with imaging the CARS beam onto the finite entrance slit of the spectrometer. Using the same 50 μm slit width used for CARS measurements and assuming an image diameter of about 100 μm at the slit plane, the corresponding slit throughput is estimated to be approximately 0.61. Taking into account the high transmission of the anti-reflection-coated collection lenses and of the short-pass filter, the overall collection efficiency for the coherent liquid-pyridine CARS signal up to the spectrometer entrance is estimated to be on the order of 50–60%.

Pump and Stokes pulse characterization

Figure S1 reports the temporal and spectral characterization of the broadband pump and Stokes pulses used for time-resolved SECARS measurements. The pulses were characterized by second-harmonic frequency-resolved optical gating (SH-FROG) after propagation through the same optical path used in the experiment. The autocorrelation traces shown in panels (a) and (c) were obtained from the measured SH-FROG maps by integrating the second-harmonic intensity over wavelength for each delay value. The spectra shown in panels (b) and (d) are the independently measured optical spectra at the sample position under the same alignment conditions.

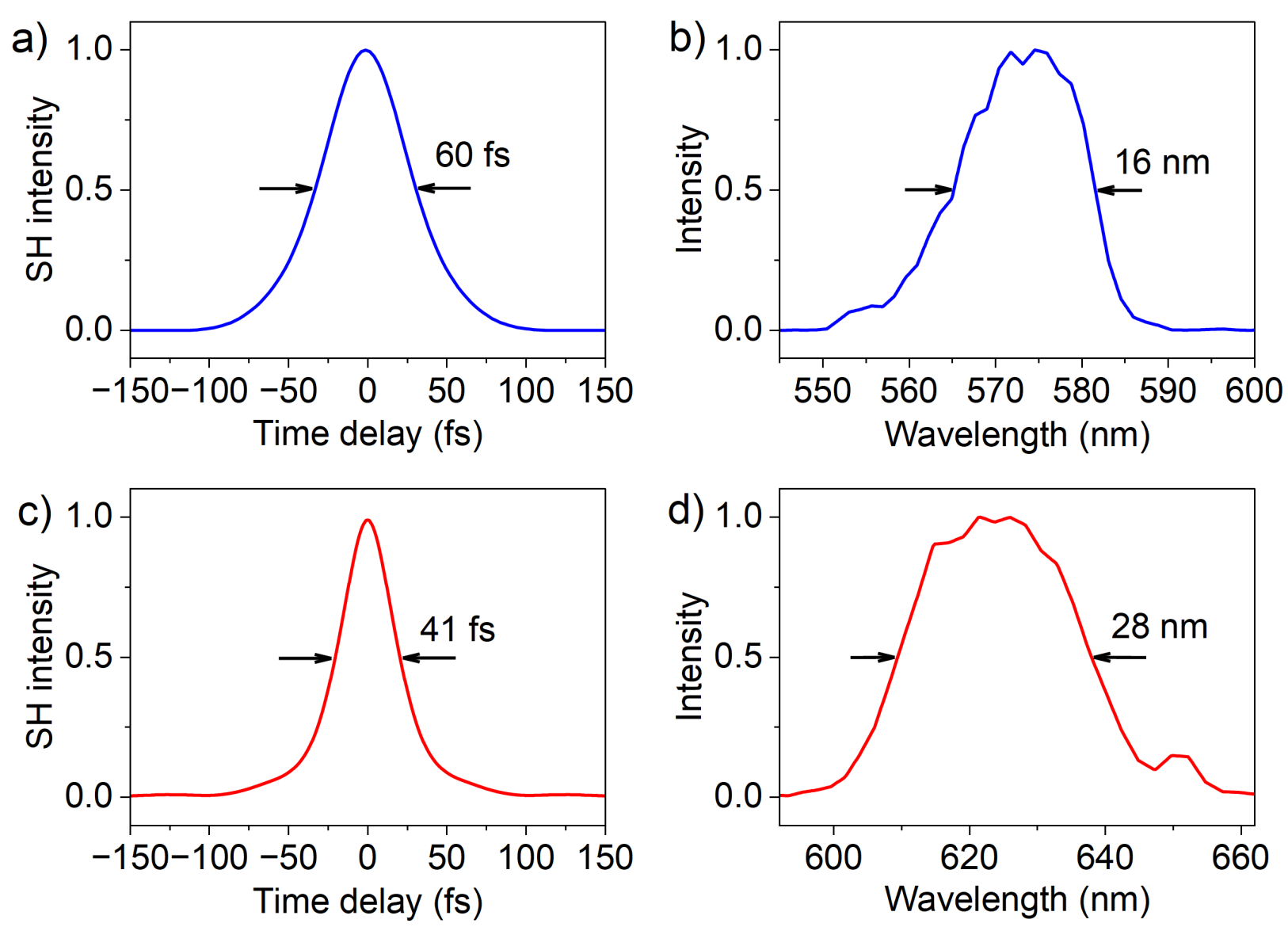

Figure S1: Temporal and spectral characterization of the pump and Stokes pulses at the sample position. (a,c) Autocorrelation traces and (b,d) measured spectra of the pump and Stokes pulses, respectively.

The pump and Stokes pulses are centered at 575 and 625 nm, with measured FWHM spectral bandwidths of 16 and 28 nm, respectively. Fourier transformation of the measured spectra after imposing a constant zero spectral phase gives transform-limited intensity FWHM durations of 35 fs for the pump and 25 fs for the Stokes pulse. The SH-FROG retrievals yield compressed pulse durations of 41 fs and 30 fs, respectively, while the corresponding autocorrelation traces have FWHM values of 60 fs and 41 fs. The main pulse parameters extracted from these measurements are summarized in Table S1, confirming that both pulses are compressed close to their transform-limited durations.

Table S1: Summary of the pump and Stokes pulse parameters measured at the sample position. The transform-limited durations were calculated by Fourier transforming the measured spectra with a constant zero spectral phase. The compressed pulse durations are obtained from the SH-FROG retrievals.

Pulse	Center wavelength (nm)	Bandwidth (nm, FWHM)	Autocorrelation (fs, FWHM)	Transform limit (fs, FWHM)	FROG duration (fs, FWHM)
Pump	575	16	60	35	41
Stokes	625	28	41	25	30

Preparation of NaDPA samples

NaDPA was prepared starting from dipicolinic acid (DPA), sodium hydroxide (NaOH), and deionized water. DPA was dissolved in an aqueous NaOH solution in order to deprotonate the carboxylic groups and form the sodium dipicolinate salt. The solution pH was kept alkaline to avoid precipitation of neutral DPA and to favor formation of the dipicolinate salt. After complete dissolution, the solution was left to evaporate under ambient conditions. The solid residue was collected and mechanically pressed to obtain a compact powder sample suitable for standoff spontaneous Raman and CARS measurements.

Wavenumber calibration of Raman, CARS, and SECARS spectra

The wavenumber axis of the Raman, CARS and SECARS spectra was calibrated using benzonitrile as a Raman reference. Benzonitrile was selected because its Raman spectrum is well known and contains intense, narrow, and well-defined vibrational bands, making it a convenient calibration target for Raman spectroscopy. Reference Raman peak positions were taken from the ChemicalBook Raman database for benzonitrile [2].

For the calibration measurement, benzonitrile was placed in a 0.5 mm-thick cuvette and measured in the same standoff CARS configuration used for the liquid-pyridine calibration. The pump, Stokes, and probe beams were temporally arranged with the probe delayed by 2.06 ps with respect to the pump/Stokes pair. At this delay, the pump and Stokes pulses coincide with a zero of the sinc^2 temporal profile of the narrowband probe, so that the instantaneous non-resonant background is strongly suppressed. Under these conditions, interference between the resonant CARS response and the non-resonant background is negligible, and the measured CARS peak positions can be directly associated with the corresponding spontaneous Raman peak positions.

The native horizontal axis of the spectrometer corresponds to the CCD pixel index, ranging from 1 to 1024 pixels. After acquisition of the delayed CARS spectrum of benzonitrile, the pixel positions of the Raman bands at 1002 and 1600 cm^{-1} were identified and assigned to their corresponding reference Raman shifts. A linear calibration between pixel index and Raman shift was then used to assign the full horizontal axis of the spectrum. This calibration was subsequently applied to all CARS and SECARS spectra, while keeping the sample position and optical alignment fixed so that the spatial origin of the coherent or spontaneous Raman signal remained unchanged.

The repeatability of the calibration procedure was better than ± 2 cm^{-1}.

Estimation of effective enhancement factors

The enhancement factors reported in the following sections are effective experimental signal ratios obtained under the specific excitation, detection, and sample-preparation conditions used in this work. They should not be interpreted as intrinsic molecule-independent quantities. In particular, for surface-deposited samples on Ag-SiNWs, the number of molecules effectively contributing to the plasmon-enhanced response depends on drying, adsorption geometry, analyte distribution, and coupling to localized electromagnetic hotspots.

Estimation of the CARS/Raman enhancement for liquid pyridine

The effective CARS/Raman enhancement of the standoff setup was estimated by comparing the spontaneous Raman and CARS signals of liquid pyridine at the 993 cm^{-1} ring-breathing mode. The spontaneous Raman spectrum was acquired using only the 515 nm probe beam, with an average power of 0.1 mW and an acquisition time of 1000 s. The CARS spectrum was acquired using pump, Stokes, and probe powers of 0.1, 0.2, and 0.1 mW, respectively, with an integration time of 10 ms.

For the spontaneous Raman spectrum, the pyridine peak at 993 cm^{-1} exhibits an amplitude of approximately 3.14×10^4 counts and a FWHM of about 24 cm^{-1}, yielding an estimated peak area $A_{\mathrm{Raman}} \simeq 7.54 \times 10^5$ counts cm^{-1}. For the CARS spectrum, the corresponding anti-Stokes peak at 993 cm^{-1} exhibits an amplitude of approximately 4.3×10^4 counts and a FWHM of about 14 cm^{-1}, corresponding to an estimated peak area $A_{\mathrm{CARS}} \simeq 6.02 \times 10^5$ counts cm^{-1}.

The two measurements were performed with different acquisition times, spectrometer slit widths, detector quantum efficiencies, and MCP gain settings. The Raman measurement was performed with a 100 μm entrance slit, while the CARS measurement used a 50 μm slit. The detector quantum efficiency was approximately 0.24 for the spontaneous Stokes Raman signal and 0.20 for the anti-Stokes CARS signal. The MCP gain corresponded to about 3000 counts per photoelectron for the Raman measurement and about 600 counts per photoelectron for the CARS measurement.

After converting the recorded counts into photoelectrons, normalizing for acquisition time and slit width, and correcting for detector quantum efficiency, the effective measured enhancement factor was calculated as

$$EF_{\mathrm{CARS/Raman}} = \frac{(A_{\mathrm{CARS}}/G_{\mathrm{CARS}})}{(A_{\mathrm{Raman}}/G_{\mathrm{Raman}})} \cdot \frac{t_{\mathrm{Raman}}}{t_{\mathrm{CARS}}} \cdot \frac{w_{\mathrm{Raman}}}{w_{\mathrm{CARS}}} \cdot \frac{\mathrm{QE}_{\mathrm{Raman}}}{\mathrm{QE}_{\mathrm{CARS}}},$$

where A is the peak area, G is the MCP conversion factor in counts per photoelectron, t is the acquisition time, w is the spectrometer slit width, and QE is the detector quantum efficiency at the corresponding wavelength. Substituting the experimental values gives

$$EF^{\mathrm{pyridine}}_{\mathrm{CARS/Raman}} \simeq 9.6 \times 10^5.$$

This value represents an effective experimental signal ratio under the specific powers, acquisition conditions, and detection settings used in the present standoff configuration.

Estimation of the CARS/Raman enhancement for compressed NaDPA powder

The effective CARS/Raman enhancement for compressed NaDPA powder was estimated from the spontaneous Raman and CARS spectra reported in Fig. 5 of the main text. The comparison was performed on the NaDPA band near 1004–1005 cm^{-1}.

For the molecule-number estimate, we assumed an effective penetration depth of 10 μm and an illuminated area of 4×10^3 $\mu\mathrm{m}^2$, giving a probed volume of approximately 4×10^{-8} cm^3. Using the measured density of the compressed NaDPA powder, 0.73 g cm^{-3}, corresponding to a molecular density of 2.08×10^{21} cm^{-3}, the number of molecules probed in the compressed powder is estimated to be

$$N_{\mathrm{NaDPA}} \simeq 8.3 \times 10^{13}.$$

For the NaDPA band at approximately 1004–1005 cm^{-1}, the spontaneous Raman peak measured in the standoff configuration has an amplitude of about 3.6×10^3 counts, while the corresponding CARS peak has an amplitude of about 4.0×10^3 counts. As for pyridine, the comparison was based on the estimated peak areas rather than on peak amplitudes. Using the measured peak amplitudes and the spectral widths associated with the corresponding spectrometer settings, we estimate $A_{\mathrm{Raman}} \simeq 8.6 \times 10^4$ counts cm^{-1} and $A_{\mathrm{CARS}} \simeq 5.6 \times 10^4$ counts cm^{-1}.

The effective CARS/Raman enhancement was calculated using the same correction procedure described above for pyridine. The detector quantum efficiencies and MCP conversion factors were kept unchanged. The parameters that differed from the pyridine measurement were the acquisition time and the excitation powers. The Raman spectrum shown in Fig. 5 of the main text was obtained by averaging ten consecutive acquisitions of 1000 s each. Since the spectra were averaged rather than summed, the relevant acquisition time for the measured peak area is $t_{\mathrm{Raman}} = 1000$ s. The CARS spectrum was acquired with an integration time of 100 ms. The average powers were 1 mW for the Raman probe beam and 0.5, 0.5, and 1 mW for the CARS pump, Stokes, and probe beams, respectively.

Applying the same correction factors used for pyridine to the peak areas reported above gives

$$EF^{\mathrm{NaDPA}}_{\mathrm{CARS/Raman}} \simeq 8 \times 10^4.$$

This value represents the effective CARS/Raman signal gain measured for compressed NaDPA powder under the present excitation conditions.

Estimation of the SECARS/CARS enhancement for Rhodamine B

The effective SECARS/CARS enhancement for Rhodamine B (RhB) was estimated by comparing the SECARS signal obtained on Ag-SiNWs with the delayed CARS signal measured from compressed RhB powder.

The excitation beams were focused onto the sample with an effective Gaussian radius $w_0 \approx 50$ μm, corresponding to an effective illuminated area of approximately

$$A_{\mathrm{eff}} \approx \frac{\pi w_0^2}{2} \approx 4 \times 10^3\ \mu\mathrm{m}^2.$$

Assuming a penetration depth of the excitation fields in the compressed RhB powder of approximately 10 μm, consistent with the multiple scattering length typically observed in strongly scattering media in backscattered CARS measurements [3], the effective interaction volume in the reference CARS measurement is estimated to be

$$V_{\mathrm{CARS}} \simeq 4 \times 10^4 \ \mu\mathrm{m}^3.$$

Considering the density and molecular mass of RhB, this interaction volume corresponds to approximately

$$N_{\mathrm{CARS}} \sim 4 \times 10^{13}$$

molecules contributing to the CARS signal. Under these conditions, the intensity of the CARS band around 1300 cm^{-1} was approximately 1.8×10^4 camera counts.

For the SECARS measurement, we consider the case in which RhB was dissolved in isopropanol at a concentration of 10^{-6} M and a droplet of approximately 1 μL was deposited onto the Ag-SiNWs substrate. The total number of molecules contained in the droplet is

$$N_{\mathrm{tot}} \sim 6 \times 10^{11}.$$

After solvent evaporation, the droplet forms an approximately circular stain with a diameter of about 6 mm, corresponding to an area of about 2.8×10^7 μm^2. Assuming that the molecules remain approximately uniformly distributed across this area, only the fraction located within the laser spot contributes to the measured SECARS signal. Using $A_{\mathrm{eff}} \simeq 4 \times 10^3$ μm^2, the number of molecules effectively probed in the SECARS experiment is estimated to be

$$N_{\mathrm{SECARS}} \sim 8 \times 10^7.$$

The SECARS signal measured at the same Raman band near 1300 cm^{-1} was approximately 3×10^3 counts.

For coherent Raman processes in homogeneous media, the anti-Stokes intensity can scale quadratically with the number of emitters because of coherent buildup along the propagation direction. This assumption is not directly applicable to nanostructured plasmonic substrates such as Ag-SiNWs. In the present case, the signal originates from spatially localized plasmonic hotspots distributed across the nanowire surface, and the radiation generated by different hotspots is expected to add predominantly incoherently. This interpretation is consistent with the approximately linear dependence of the SECARS signal on molecular concentration observed in Fig. 7(b) of the main text.

Since the reference CARS and SECARS measurements were performed with different excitation powers, the comparison was normalized to the product of pump, Stokes, and probe powers. Under these assumptions, the effective enhancement factor was calculated as

$$EF_{\mathrm{SECARS/CARS}} = \frac{I_{\mathrm{SECARS}}}{I_{\mathrm{CARS}}} \cdot \frac{P_{\mathrm{tot}}^{\mathrm{CARS}}}{P_{\mathrm{tot}}^{\mathrm{SECARS}}} \cdot \frac{N_{\mathrm{CARS}}}{N_{\mathrm{SECARS}}},$$

where

$$P_{\mathrm{tot}} = P_{\mathrm{pump}} P_{\mathrm{Stokes}} P_{\mathrm{probe}}.$$

For the SECARS measurements on Ag-SiNWs, the pump, Stokes, and probe powers were 20, 40, and 10 μW, respectively. For the reference CARS measurement on the pressed RhB tablet, the corresponding powers were 100, 200, and 100 μW. Substituting these values gives

$$EF^{\mathrm{RhB}}_{\mathrm{SECARS/CARS}} \simeq 2 \times 10^7.$$

Because RhB is measured under partially resonant conditions, this value represents an effective substrate-induced enhancement specific to the present RhB experiment.

Estimation of the SECARS/CARS enhancement for NaDPA

The effective SECARS/CARS enhancement for NaDPA was estimated by comparing the SECARS signal at 10^{-5} M with the reference CARS signal measured from compressed NaDPA powder.

For the CARS measurement, the pump, Stokes, and probe powers were 0.5, 0.5, and 1 mW, respectively, the integration time was 100 ms, and the peak amplitude of the 1005 cm^{-1} band was approximately 4.0×10^3 counts. For the SECARS measurement at 10^{-5} M, the corresponding peak at 1006.7 cm^{-1} had an amplitude of approximately 1.8×10^3 counts with an integration time of 1 s.

Assuming the same effective laser area used above, $A_{\text{laser}} \simeq 4 \times 10^3\ \mu\text{m}^2$, and a dried droplet stain area of about $2.8 \times 10^7\ \mu\text{m}^2$, the number of NaDPA molecules probed in the SECARS measurement at 10^{-5} M is estimated to be

$$N_{\text{SECARS}} \simeq 8.6 \times 10^8.$$

The number of molecules contributing to the reference CARS signal from compressed NaDPA powder was estimated above as

$$N_{\text{CARS}} \simeq 8.3 \times 10^{13}.$$

Using the same SECARS/CARS enhancement definition adopted for RhB, with the detected signals normalized to their respective integration times and to the product of pump, Stokes, and probe powers, the effective enhancement factor is

$$EF_{\text{SECARS/CARS}} = \frac{I_{\text{SECARS}}}{I_{\text{CARS}}} \cdot \frac{P_{\text{tot}}^{\text{CARS}}}{P_{\text{tot}}^{\text{SECARS}}} \cdot \frac{N_{\text{CARS}}}{N_{\text{SECARS}}},$$

where

$$P_{\text{tot}} = P_{\text{pump}} P_{\text{Stokes}} P_{\text{probe}}.$$

For the NaDPA measurements,

$$\frac{I_{\text{SECARS}}}{I_{\text{CARS}}} = \frac{1.8 \times 10^3/1\ \text{s}}{4.0 \times 10^3/0.1\ \text{s}},$$

and

$$\frac{P_{\text{tot}}^{\text{CARS}}}{P_{\text{tot}}^{\text{SECARS}}} = \frac{0.5 \times 0.5 \times 1}{0.02 \times 0.04 \times 0.01}.$$

Substituting these values gives

$$EF_{\text{SECARS/CARS}}^{\text{NaDPA}} \simeq 1.4 \times 10^8.$$

This value represents the effective SECARS/CARS enhancement measured for NaDPA under the present deposition and excitation conditions.

Concentration dependence of standoff SECARS detection of Rhodamine B and NaDPA

At low concentrations the SECARS intensity exhibits an approximately linear dependence on the molecular concentration, consistent with the expected increase in the number of molecules contributing to the nonlinear polarization. In the present plasmonic substrate, however, the SECARS response is generated by spatially localized electromagnetic hotspots distributed across the nanowire surface. Because the radiation emitted by different hotspots adds predominantly incoherently, the signal is expected to scale approximately linearly with the number of molecules effectively coupled to the plasmonic nanostructure, as experimentally observed at low concentrations in Fig. 7(b).

At higher concentrations a deviation from the linear trend becomes visible. This behavior likely reflects a change in the relative weight of different molecular populations on the substrate. At low concentration, the detected SECARS signal is dominated by molecules located in the highest-enhancement hotspot regions, where the local plasmonic field strongly amplifies the coherent Raman response. As the deposited amount increases, additional molecules are not necessarily placed in these highest-enhancement regions, but may instead occupy lower-enhancement regions, form aggregates or multilayers, or remain farther from the metal surface. These molecules can still absorb the excitation fields and contribute to fluorescence or weak coherent Raman background, but they experience a much lower plasmonic enhancement than molecules directly located in hotspots. As a result, the marginal SECARS contribution of additional molecules becomes progressively smaller, leading to an apparent sub-linear concentration dependence.

A similar behavior was previously observed for Rhodamine 6G on Ag-decorated SiNWs, where the SERS signal increased linearly only over a limited concentration range and then decreased at higher concentrations. This was attributed to saturation of SERS-active sites and to the increasing contribution of non-adsorbed dye molecules, which enhance the fluorescence background and absorb part of the incident radiation [4]. In the present SECARS

measurements, analogous effects, together with local field screening, quenching, drying-induced redistribution, and possible weak coherent buildup in more densely populated low-enhancement regions, may contribute to the observed deviation from linearity.

As for RhB, the deviation from a linear concentration dependence for the SECARS intensity of NaDPA should not be interpreted as simple saturation of all plasmonic hotspots. In the case of NaDPA, which does not exhibit a strong visible fluorescence background under the present excitation conditions, the deviation is more likely associated with the morphology of the dried salt deposit and with the redistribution of the analyte relative to the plasmonic hotspots.

At higher concentrations, NaDPA can form more non-uniform dried residues, local aggregates, or crystallites. Additional molecules are therefore not necessarily placed in the highest-enhancement regions, but may occupy lower-enhancement regions or be located farther from the Ag surface, where the plasmonic amplification is much weaker. These molecules can still contribute to a weak coherent Raman response, possibly including local coherent buildup in more densely populated regions, but with a much lower effective enhancement than molecules located directly in plasmonic hotspots. As a result, the marginal SECARS contribution of additional molecules becomes progressively smaller, producing an apparent sub-linear dependence at higher concentration.

Assignment of the NaDPA SECARS bands

To further assess the spectral assignment, Fig. 10 compares the SECARS spectrum of NaDPA deposited on Ag-SiNWs with a spontaneous Raman spectrum of dipicolinic acid (DPA) taken from Ref. [5]. This comparison is appropriate because, in aqueous solution, NaDPA is dissociated and sodium is expected to act mainly as a solvated counterion. The vibrational response detected in SECARS is therefore attributed to the dipicolinate/DPA molecular framework adsorbed in the vicinity of the Ag nanostructured surface, rather than to sodium itself.

The SECARS spectrum preserves the main fingerprint of DPA/dipicolinate. The intense band observed at about 1007 cm^{-1} is assigned to the symmetric breathing/stretching mode of the pyridine ring, which is one of the most characteristic Raman markers of DPA and dipicolinate salts. In the higher-frequency region, the bands around 1380–1400 cm^{-1} and 1420–1440 cm^{-1} are mainly associated with the symmetric OCO stretching of the carboxylate groups and ring C–H bending or C–C stretching modes, while the bands around 1560–1580 cm^{-1} are assigned to asymmetric OCO stretching and pyridine-ring stretching modes.

The SECARS spectrum does show variations of relative intensities and peak positions with respect to the spontaneous Raman spectrum. This behavior is expected for surface-enhanced measurements of DPA-like species on silver. Previous SERS studies of DPA showed that metal–molecule interactions can shift vibrational frequencies and selectively enhance different modes, making the assignment of SERS bands less straightforward than for ordinary Raman spectra[6, 7]. In particular, the carboxylate groups and the pyridine ring can interact with the Ag surface, and their vibrational modes can be enhanced differently depending on adsorption geometry, molecular orientation, and local electromagnetic field distribution at the plasmonic hotspots.

The strongest SECARS contribution at 1007 cm^{-1} is consistent with the prominent DPA ring-breathing mode reported in Raman and SERS studies. The relative enhancement of the bands in the 1380–1450 cm^{-1} range indicates a significant contribution from carboxylate-related vibrations, suggesting that the dipicolinate species interacts with the Ag surface at least partly through the carboxylate groups. The modifications observed in the 1550–1600 cm^{-1} region can be attributed to changes in the relative enhancement of asymmetric OCO stretching and ring-stretching modes. Therefore, the spectral differences between spontaneous Raman and SECARS should be interpreted as the expected consequence of adsorption on a plasmonic metallic substrate and mode-dependent surface enhancement.

Spectral comparison between Raman, CARS, and SECARS spectra of Rhodamine B

The spectral fidelity of the RhB SECARS signal was analyzed by comparing the SECARS spectrum obtained from RhB deposited on Ag-SiNWs with the spontaneous Raman spectrum of pure RhB powder and with a time-delayed CARS spectrum acquired from compressed RhB powder without the plasmonic substrate, as shown in Fig. 8 of the main text. The CARS spectrum was acquired at the same probe delay of 2.06 ps used for the SECARS measurements. This comparison makes it possible to distinguish spectral modifications associated with the coherent Raman process itself from those induced by the interaction of the molecules with the plasmonic Ag-SiNWs substrate.

The delayed CARS spectrum of compressed RhB powder reproduces the main vibrational bands observed in the spontaneous Raman reference spectrum, with no major spectral shifts. This indicates that, under delayed-probe

conditions, the CARS process itself does not introduce substantial shifts of the Raman bands. This behavior is consistent with the time-resolved detection scheme used here: by delaying the probe pulse to a zero of its sinc^2 temporal profile, the instantaneous non-resonant background is strongly suppressed, and the measured CARS spectrum is dominated by the resonant vibrational response. In contrast, at zero delay, as shown in Fig. 6(a) of the main text, interference between the resonant and non-resonant contributions can distort the apparent spectral lineshapes and produce asymmetric or dispersive features.

Larger differences are instead observed when comparing the spontaneous Raman and delayed CARS spectra with the SECARS spectrum acquired on Ag-SiNWs. In particular, some bands exhibit shifts larger than the instrumental spectral resolution, and the relative peak intensities are substantially modified. These differences are attributed primarily to the interaction between RhB molecules and the Ag-decorated nanowire substrate. In SECARS, the detected signal arises from molecules located in the vicinity of plasmonic hotspots, where adsorption on the metallic surface, local molecular orientation, and the heterogeneous electromagnetic environment can modify both the vibrational frequencies and the relative enhancement of different Raman modes. In addition, the partially resonant character of RhB under the present excitation conditions can contribute to mode-dependent changes in relative intensities, because resonance Raman processes may preferentially enhance specific vibrational modes coupled to the electronic transition.

The same effects can also contribute to the residual asymmetries observed in some SECARS bands. Although the delayed-probe scheme strongly suppresses the instantaneous non-resonant contribution, it does not necessarily remove all sources of lineshape modification in resonant molecules adsorbed on plasmonic substrates. Small residual asymmetries may therefore arise from a combination of residual non-resonant background, mode-dependent vibrational dephasing, the partially resonant character of RhB, and molecule–substrate interactions at the Ag-SiNWs hotspots. This interpretation is consistent with previous SERS studies on Ag-decorated SiNWs, where the SERS response was shown to depend strongly on the size, spatial distribution, and interparticle distance of the Ag nanoparticles, which determine the localized surface plasmon resonances, hotspot density, and mode-dependent electromagnetic enhancement [4].

Spatial uniformity of the SECARS response

The spatial reproducibility of the SECARS response was evaluated by acquiring spectra at different positions on the same Ag-SiNWs substrate after deposition of RhB at a concentration of 10^{-5} M. This concentration was selected because it provides a clear SECARS signal while remaining within the concentration range investigated in the main manuscript. The measurements were performed under the same experimental conditions used for the concentration-dependent SECARS measurements reported in the main text.

Figure S2 shows ten SECARS spectra acquired at different positions along the vertical direction of the sample. The black curve represents the average spectrum, while the shaded region indicates ± 1 standard deviation at each Raman shift. The overall spectral shape is preserved across the investigated positions, with moderate variations in signal amplitude. This indicates that the Ag-SiNWs substrate provides a reasonably uniform distribution of plasmonic enhancement sites over the probed region.

To quantify the spatial variability, the integrated SECARS intensities of the peaks at 1178.1 and 1282.6 cm^{-1} were calculated over a spectral interval of ± 10 cm^{-1} around each peak for all sampled positions. The corresponding values are reported in the inset of Fig. S2. The observed variations are compatible with the intrinsic spatial heterogeneity expected for plasmonic nanostructured substrates and confirm that the SECARS response remains reproducible over different positions of the Ag-SiNWs surface. This level of spatial uniformity is relevant for standoff measurements, where small variations in the excitation spot position on the substrate can occur during alignment or remote interrogation.

Scaling of the SECARS signal with working distance

In the present backscattering SECARS geometry, the detected signal is expected to scale approximately with the collection solid angle. For a fixed collection aperture, this gives an approximate $1/r^2$ dependence on the working distance r. Therefore, increasing the working distance for example from 20 cm to 1 m would reduce the detected signal by a factor

$$G = \left(\frac{100\ \text{cm}}{20\ \text{cm}}\right)^2 = 25,$$

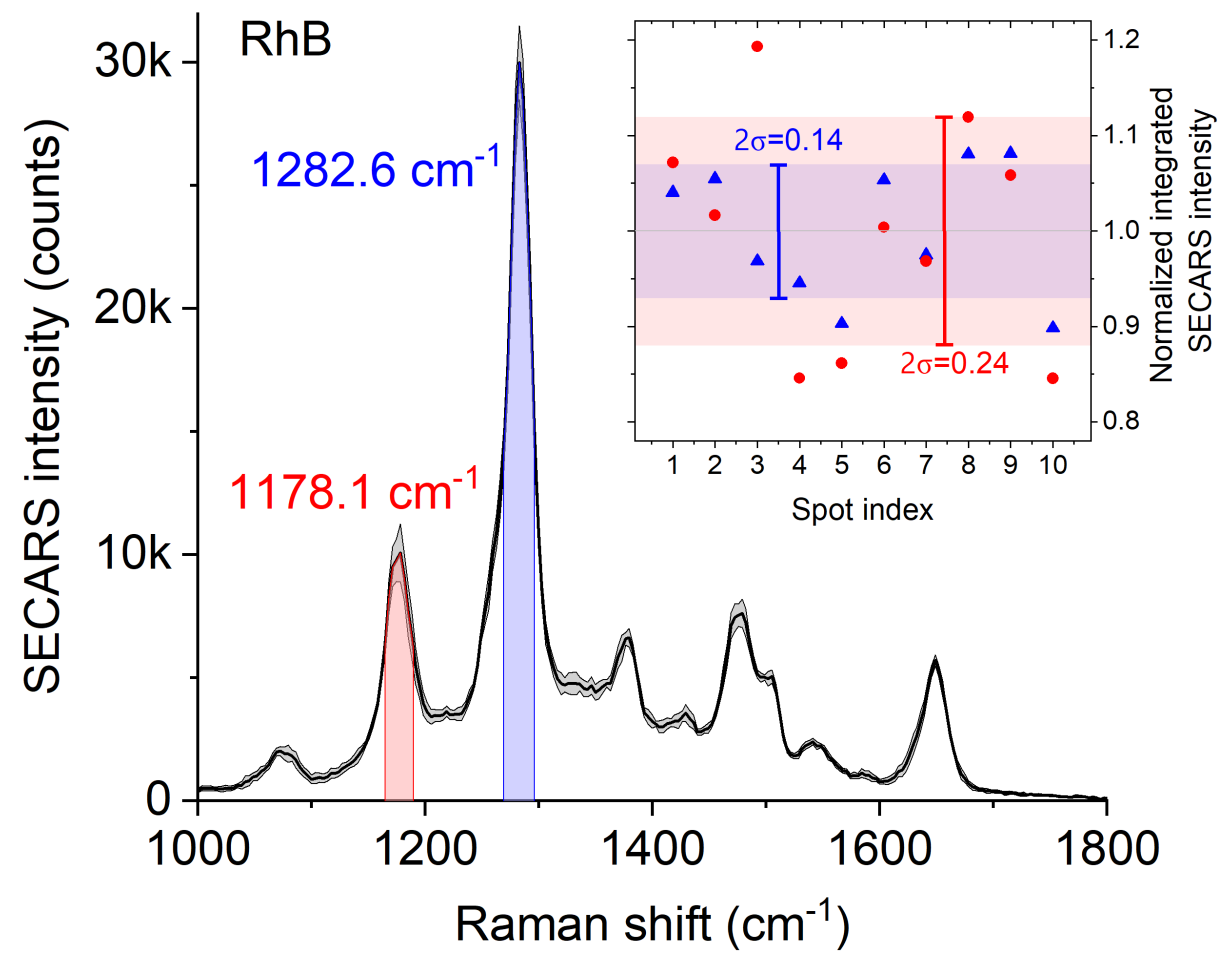

Figure S2: Assessment of the spatial uniformity of the SECARS response for a Rhodamine B concentration of 10^{-5} M. Experimental conditions are the same as those used for the Rhodamine B concentration-dependent SECARS measurements reported in the main text. Ten spectra were acquired at different positions along the vertical direction of the sample. The black curve represents the mean SECARS spectrum, while the shaded region corresponds to ± 1 standard deviation at each Raman shift. Inset: Integrated SECARS intensity of the peaks at 1178.1 cm^{-1} and 1282.6 cm^{-1}, calculated over a spectral interval of ± 10 cm^{-1} around each peak for the ten sampled positions. The colored bands represent ± 1 standard deviation of the integrated peak areas.

unless compensated by a larger collection aperture, higher excitation energies, longer integration times, or a larger illuminated substrate area.

Because SECARS is a third-order nonlinear process, the generated signal depends on the product of pump, Stokes, and probe intensities. If the illuminated area is kept fixed and no saturation or damage occurs, a geometrical loss factor G could in principle be compensated by increasing each of the three excitation powers by approximately $G^{1/3}$. For the 20 cm to 1 m case, this gives

$$G^{1/3} = 25^{1/3} \simeq 2.9.$$

Thus, in an ideal fixed-spot geometry, operation at 1 m would require increasing each beam power by about a factor of 3. This approach, however, increases the fluence on the plasmonic substrate and is ultimately limited by substrate damage and possible photothermal effects.

A more conservative strategy is to increase the illuminated area while keeping the fluence approximately constant. In this case, compensation of the same geometrical loss requires increasing the illuminated area by the factor G, corresponding to an increase of the beam diameter by $G^{1/2}$. For the 20 cm to 1 m case, this corresponds to a fivefold increase in beam diameter. Starting from a beam diameter of about 100 μm in the present experiment, this would correspond to a diameter of about 500 μm at 1 m.

Since the working distance is also increased by a factor of five, the angular size of the illuminated region remains approximately unchanged. Consequently, the size of the image formed at the spectrometer entrance is not expected to increase with respect to the present 20 cm configuration. Under this constant-fluence scaling, coupling through the spectrometer slit is therefore not expected to become the primary limitation. The practical limitations are instead set by the available pulse energies, the damage threshold of the plasmonic substrate, the usable substrate area, and the collection aperture.

This estimate is consistent with our previous field-deployable standoff CARS implementation, where operation at a 1 m working distance was achieved by using higher pulse energies and a larger beam diameter on the target [8]. In that work, NaDPA powder was measured at 1 m with pulse energies of 4, 4, and 3 μJ for the pump, Stokes, and probe beams, respectively, a beam diameter of about 360 μm, and a 100 ms acquisition time. Although the present experiment involves SECARS on a plasmonic substrate rather than conventional CARS from a powder target, the same geometrical scaling applies to the collection efficiency and to the trade-off between pulse energy, illuminated area, and fluence.

Comparison with literature enhancement factors

Table S2 reports representative enhancement factors from the literature for SERS, CARS, and SECARS measurements, together with the effective enhancement factors estimated in the present work. The comparison is intended to provide an order-of-magnitude context for the signal amplification achieved in the standoff SECARS configuration.

It should be noted that enhancement factors reported in different studies are not always defined in the same way. Depending on the experiment, they may refer to SERS/Raman, CARS/Raman, SECARS/CARS, or overall SECARS/Raman signal ratios, and they can also depend on excitation powers, acquisition conditions, molecular coverage, substrate morphology, and the estimated number of probed molecules. For this reason, the values in Table S2 should be interpreted as representative literature benchmarks rather than as directly comparable universal quantities.

Table S2: Comparison of representative enhancement factors reported in the literature for SERS, CARS, and SECARS experiments. Here, $EF_{\mathrm{SERS}} \equiv EF_{\mathrm{SERS/Raman}}$, $EF_{\mathrm{CARS}} \equiv EF_{\mathrm{CARS/Raman}}$, $EF_{\mathrm{SECARS/CARS}}$ denotes the enhancement of SECARS relative to conventional CARS, and $EF_{\mathrm{SECARS}} \equiv EF_{\mathrm{SECARS/Raman}}$.

Ref.	Tech.	Geom.	$\mathrm{EF_{SERS}}$	$\mathrm{EF_{CARS}}$	$\mathrm{EF_{SECARS/CARS}}$	$\mathrm{EF_{SECARS}}$
[9]	SERS	micro	10^6–10^{12}			
[10]	SERS	micro	2.3×10^8			
[11]	SERS	micro	4.12×10^9			
[12]	SERS	micro	$\sim 10^6$			
[13]	SERS	micro	10^5–10^{10}			
[14]	CARS	micro		$\sim 10^7$		
[15]	CARS	micro		$\sim 10^6$		
[16]	SECARS	micro			$\sim 10^5$	$\sim 10^9$–10^{10}
[17]	SECARS	standoff cm scale			10^7	
[18]	SECARS	micro			$> 10^9$	
[19]	SECARS	micro			$\sim 10^9$	
[20]	SECARS	micro				$\sim 10^{11}$
This work, pyridine/RhB	SECARS	standoff 20 cm		9.6×10^5	2×10^7	–
This work, NaDPA	SECARS	standoff 20 cm		8×10^4	1.4×10^8	$\sim 10^{13}$

References

[1] International Organization for Standardization. ISO 11146:2005, Lasers and laser-related equipment − Test methods for laser beam widths, divergence angles and beam propagation ratios. International Standard, 2005.

[2] ChemicalBook. `https://www.chemicalbook.com/SpectrumEN_100-47-0_Raman.htm` (accessed 2026-09-11).

[3] Yu Huang, Andrew Soroka, Kenneth Cohen, and Arthur Dogariu. Backscattered coherent anti-stokes raman scattering for at range detection of dipicolinic acid and four-wave mixing multiple scattering. *Journal of Modern Optics*, 54:2473–2480, 11 2007.

[4] Cristiano D'Andrea, Maria J.Lo Faro, Giulia Bertino, Paolo M. Ossi, Fortunato Neri, Sebastiano Trusso, Paolo Musumeci, Matteo Galli, Nicola Cioffi, Alessia Irrera, Francesco Priolo, and Barbara Fazio. Decoration of silicon nanowires with silver nanoparticles for ultrasensitive surface enhanced raman scattering. *Nanotechnology*, 27:375603, 8 2016.

[5] Lingbo Kong, Peter Setlow, and Yong-qing Li. Analysis of the raman spectra of ca2+-dipicolinic acid alone and in the bacterial spore core in both aqueous and dehydrated environments. *Analyst*, 137(16):3683–3689, 08 2012.

[6] Steven E. J. Bell, Joseph N. Mackle, and Narayana M. S. Sirimuthu. Quantitative surface-enhanced raman spectroscopy of dipicolinic acid—towards rapid anthrax endospore detection. *Analyst*, 130(4):545–549, 01 2005.

[7] Stuart Farquharson, Chetan Shende, Alan Gift, and Frank Inscore. Detection of bacillus spores by surface-enhanced raman spectroscopy. In Stephen A. Morse, editor, *Bioterrorism*, chapter 2. IntechOpen, London, 2012.

[8] Nicola Coluccelli, Gianluca Galzerano, Paolo Laporta, Kelly Curtis, Claire L. Lonsdale, Debbie Padgen, Christopher R. Howle, and Giulio Cerullo. Standoff detection of bacterial spores by field deployable coherent raman spectroscopy. *Scientific Reports*, 13:2634, 12 2023.

[9] Katrin Kneipp, Harald Kneipp, Irving Itzkan, Ramachandra R Dasari, and Michael S Feld. Surface-enhanced raman scattering and biophysics. *Journal of Physics: Condensed Matter*, 14(18):R597, apr 2002.

[10] Elisabeth Galopin, Jacques Barbillat, Yannick Coffinier, Sabine Szunerits, Gilles Patriarche, and Rabah Boukherroub. Silicon nanowires coated with silver nanostructures as ultrasensitive interfaces for surface-enhanced raman spectroscopy. *ACS Applied Materials and Interfaces*, 1:1396–1403, 7 2009.

[11] Xuemei Han, Hui Wang, Xuemei Ou, and Xiaohong Zhang. Highly sensitive, reproducible, and stable sers sensors based on well-controlled silver nanoparticle-decorated silicon nanowire building blocks. *Journal of Materials Chemistry*, 22:14127–14132, 7 2012.

[12] Jian An Huang, Ying Qi Zhao, Xue Jin Zhang, Li Fang He, Tai Lun Wong, Ying San Chui, Wen Jun Zhang, and Shuit Tong Lee. Ordered ag/si nanowires array: Wide-range surface-enhanced raman spectroscopy for reproducible biomolecule detection. *Nano Letters*, 13:5039–5045, 11 2013.

[13] Ioannis Kochylas, Spiros Gardelis, Vlassis Likodimos, Konstantinos P. Giannakopoulos, Polycarpos Falaras, and Androula G. Nassiopoulou. Improved surface-enhanced-raman scattering sensitivity using si nanowires/silver nanostructures by a single step metal-assisted chemical etching. *Nanomaterials*, 11, 7 2021.

[14] Wei Min, Christian W. Freudiger, Sijia Lu, and X. Sunney Xie. Coherent nonlinear optical imaging: Beyond fluorescence microscopy. *Annual Review of Physical Chemistry*, 62(Volume 62, 2011):507–530, 2011.

[15] Georgi I. Petrov, Rajan Arora, Vladislav V. Yakovlev, Xi Wang, Alexei V. Sokolov, and Marlan O. Scully. Comparison of coherent and spontaneous raman microspectroscopies for noninvasive detection of single bacterial endospores. *Proceedings of the National Academy of Sciences*, 104:7776–7779, 5 2007.

[16] Christian Steuwe, Clemens F. Kaminski, Jeremy J. Baumberg, and Sumeet Mahajan. Surface enhanced coherent anti-stokes raman scattering on nanostructured gold surfaces. *Nano Letters*, 11:5339–5343, 12 2011.

[17] Dmitri V. Voronine, Alexander M. Sinyukov, Xia Hua, Kai Wang, Pankaj K. Jha, Elango Munusamy, Steven E. Wheeler, George Welch, Alexei V. Sokolov, and Marlan O. Scully. Time-resolved surface-enhanced coherent sensing of nanoscale molecular complexes. *Scientific Reports*, 2:891, 2012.

[18] Anton D. Shutov, Zhenhuan Yi, Jizhou Wang, Alexander M. Sinyukov, Zhe He, Chenwei Tang, Jiahao Chen, Esther J. Ocola, Jaan Laane, Alexei V. Sokolov, Dmitri V. Voronine, and Marlan O. Scully. Giant chemical surface enhancement of coherent raman scattering on mos 2. *ACS Photonics*, 5:4960–4968, 12 2018.

[19] Shamsul Abedin, Yong Li, Abid Anjum Sifat, Khokan Roy, and Eric O. Potma. Enhancement of molecular coherent anti-stokes raman scattering with silicon nanoantennas. *Nano Letters*, 22:6685–6691, 8 2022.

[20] Yu Zhang, Yuan-Ru Zhen, Oara Neumann, Jesse K. Day, Peter Nordlander, and Naomi J. Halas. Coherent anti-stokes raman scattering with single-molecule sensitivity using a plasmonic fano resonance. *Nature Communications*, 5:4424, 2014.
